# Influence of surface atomic structure demonstrated on oxygen incorporation mechanism at a model perovskite oxide

**DOI:** 10.1038/s41467-018-05685-5

**Published:** 2018-09-13

**Authors:** Michele Riva, Markus Kubicek, Xianfeng Hao, Giada Franceschi, Stefan Gerhold, Michael Schmid, Herbert Hutter, Juergen Fleig, Cesare Franchini, Bilge Yildiz, Ulrike Diebold

**Affiliations:** 1 Institute of Applied Physics, TU Wien, Wiedner Hauptstraβe 8-10/E134, 1040 Wien, Austria; 2 Institute of Chemical Technologies and Analytics, TU Wien, Getreidemarkt 9/164EC, 1060 Wien, Austria; 3 Key Laboratory of Applied Chemistry, Department of Chemical Engineering, Yanshan University, 066004 Qinhuangdao, China; 4 Faculty of Physics and Center for Computational Materials Science, University of Vienna, Sensengasse 8/8, 1090 Vienna, Austria; 5 Laboratory for Electrochemical Interfaces, Departments of Nuclear Science and Engineering, and Materials Science and Engineering, Massachusetts Institute of Technology, 77 Massachusetts Avenue, Cambridge, MA 02139 USA

## Abstract

Perovskite oxide surfaces catalyze oxygen exchange reactions that are crucial for fuel cells, electrolyzers, and thermochemical fuel synthesis. Here, by bridging the gap between surface analysis with atomic resolution and oxygen exchange kinetics measurements, we demonstrate how the exact surface atomic structure can determine the reactivity for oxygen exchange reactions on a model perovskite oxide. Two precisely controlled surface reconstructions with (4 × 1) and (2 × 5) symmetry on 0.5 wt.% Nb-doped SrTiO_3_(110) were subjected to isotopically labeled oxygen exchange at 450 °C. The oxygen incorporation rate is three times higher on the (4 × 1) surface phase compared to the (2 × 5). Common models of surface reactivity based on the availability of oxygen vacancies or on the ease of electron transfer cannot account for this difference. We propose a structure-driven oxygen exchange mechanism, relying on the flexibility of the surface coordination polyhedra that transform upon dissociation of oxygen molecules.

## Abstract (web summary)

The availability of surface oxygen vacancies or electrons is often viewed as the defining factor for the reactivity of perovskite oxides. Two precisely controlled surfaces on SrTiO_3_(110) show strikingly different O exchange kinetics, which the authors ascribe to the flexibility of the surface polyhedra.

## Introduction

Oxygen reduction and oxygen evolution often limit the efficiency of energy conversion technologies including fuel cells, electrolyzers, and photo-/electrochemical water splitting. Perovskite oxides (of unit formula ABO_3_) are widely used and studied materials for enabling these reactions at elevated temperatures. They are used in solid oxide fuel cells (SOFC) for electricity production^1–3^, in the synthesis of fuels by electrolysis of water or steam^4^ and in the thermochemical splitting of water and CO_2_^5^. The reactivity on these perovskite oxides is often interpreted in terms of the availability of surface oxygen vacancies (V_O_)^6–10^ or electrons^11–15^ and the position of the oxygen 2*p* band center^16^. Undoubtedly, the atomic-scale details of surface structures ought to also be critical in determining the speed of the oxygen reduction and evolution reactions (ORR and OER), which is measured either electrochemically or by incorporation of isotopically labeled ^18^O^3,17,18^. Intriguingly, none of these canonical reactivity models consider the role of the precise surface atomic structure. The important question is how the atomic configuration at the surface affects the ORR/OER mechanisms at the molecular level, either via these reactivity-determining factors or directly via the structure itself. For example, it was shown that La_0.7_Sr_0.3_MnO_3_^19^, La_2_NiO_4_^20^, SrRuO_3_^21^, and SrTiO_3_^22^ possess different oxygen exchange and water splitting kinetics depending on the surface crystallographic orientation, but the actual atomic structure of the surfaces was not resolved. Reliable computational modeling of surface reactivity at the first-principles level necessarily requires the geometric positions of the surface atoms as an input, which, in turn, need to be confirmed by experiments.

Knowing the geometric arrangement of the surface atoms under reaction conditions has been highly difficult, however. There are scarcely any methods that can determine the surface structure and measure the reactivity to oxygen exchange without perturbing the surface structure under reaction conditions of elevated temperatures and realistic reactant pressures. In addition, many of the Sr-doped perovskite oxides that are used in electrocatalytic or thermochemical reactions [e.g., La_0.8_Sr_0.2_MnO_3_ (LSM)^17^, La_0.6_Sr_0.4_CoO_3_ (LSC)^23^, La_0.6_Sr_0.4_Co_0.2_Fe_0.8_O_3_ (LSCF)^24^, and Ba_0.5_Sr_0.5_Co_0.8_Fe_0.2_O_3_ (BSCF)^25^] segregate out Sr-rich-insulating phases^26–30^, and it is impossible to resolve these highly heterogeneous surface regions with atomic resolution.

In the present study, we marry physical surface science studies with kinetic oxygen exchange measurements on precisely controlled atomic structures and demonstrate how these affect oxygen exchange mechanisms and kinetics. We take SrTiO_3_ as a prototypical model perovskite oxide, primarily due to our ability to prepare SrTiO_3_(110) with two distinctly different and controllable surface phases with solved structures^31^. Another advantage of this system is the fact that Sr segregation is suppressed if single-crystal SrTiO_3_ surfaces are stabilized by a reconstruction^32^. We use 0.5 wt.% Nb-doped samples that are sufficiently conductive for evaluating the atomic structure with STM. The relevant bulk V_O_ concentration expected under the experimental conditions of this work is extremely low, as discussed later. The bulk oxygen transport is thus strongly suppressed, but the surface oxygen exchange reaction can still be probed in isotope exchange experiments. By quantifying the ^18^O exchange for these two reconstructions, while keeping all other experimental parameters exactly constant, we find that their reactivity differs by a factor of three. Density functional theory (DFT) calculations on these precisely resolved surface structures reveal that this difference is neither related to oxygen vacancies nor to variations in work function or surface potential that would affect the availability of electrons on this material. Instead, the structural details determine the interaction with the molecular oxygen. Our results reveal the polyhedral flexibility up to the ideal coordination limit as an important and previously unexplored factor that can govern the reactivity to oxygen exchange reactions on perovskite oxides surfaces.

## Results

### Characterization of SrTiO_3_(110) surface reconstructions

**Fig. 1:**
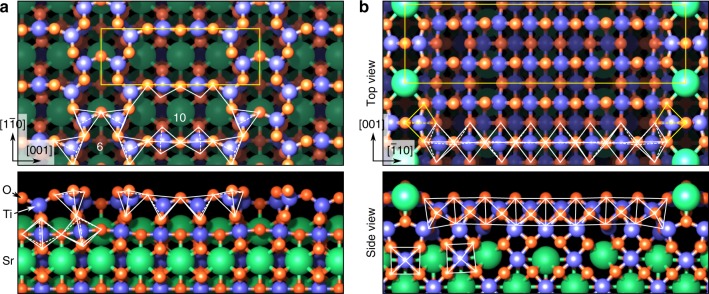
Surface structure models on SrTiO_3_(110). **a** SrTiO_3_(110)–(4 × 1), and **b** SrTiO_3_(110)–(2 × 5) surface reconstructions^31^. Top view (top) and cross-section view (bottom). Notice that the structures are displayed with a 90° in-plane rotation. Ti, Sr, and O atoms are drawn as blue, green, and red spheres, respectively. The Ti coordination polyhedra are represented by white lines. While the (4 × 1) surface (**a**) is composed of ten- and six-membered rings of corner-sharing tetrahedra, the (2 × 5) surface (**b**) consists of a bilayer of octahedra

Figure 1 shows the (4 × 1)- and (2 *×* 5)-reconstructed surfaces of SrTiO_3_(110), prepared as described in the “Methods” section. In both cases, a layer of TiO_*x*_ polyhedra (*x* = 4, 5, 6 as defined below) sits on a (SrTiO)^4+^ plane, which is only marginally distorted from its bulk structure. These top titania overlayers have distinctly different structural properties, however. The (4 × 1) surface (Fig. 1a) consists of a porous network of corner-sharing tetrahedrally coordinated TiO_4_ units, arranged into six- and ten-membered rings (highlighted by tetrahedra in Fig. 1a). The SrTiO_3_(110)*-*(2 *×* 5)-reconstructed surface (Fig. 1b) consists, instead, of a bilayer of octahedrally coordinated Ti atoms. The subsurface layer consists of edge- and corner-sharing octahedra. The topmost surface layer hosts 16 edge-sharing TiO_6_ octahedra, and two TiO_5_ units, in which the apical oxygen atom is missing. Finally, fivefold-coordinated Sr atoms alternate with the TiO_5_ units, with a twofold periodicity along [001]. Similar to other surfaces of SrTiO_3_^33^, these reconstructions on n-type SrTiO_3_(110) form due to thermodynamic conditions, and in this case mainly due to the minimization of surface energy^31^ as a function of chemical potential of Ti and Sr, and of strain energy^34^. The same reconstructions exist on undoped or p-type-doped SrTiO_3_(110) surfaces.

**Fig. 2:**
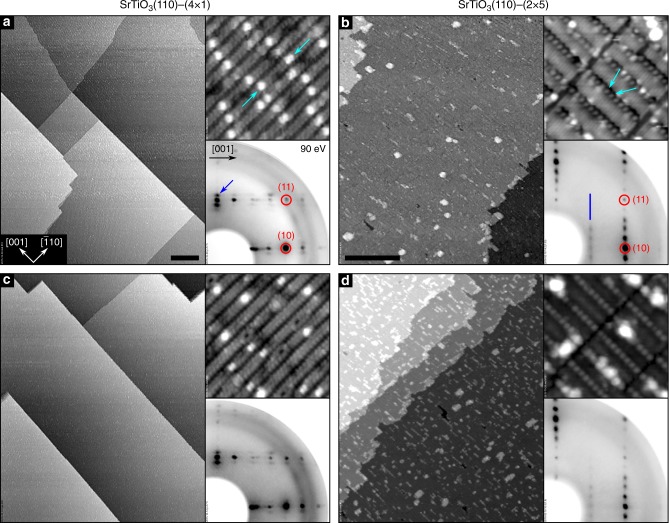
Surface structures of SrTiO_3_(110), and their stability upon treatment in oxygen atmosphere. Scanning tunneling microscopy (STM) images (main panels: **a**, **c** 410 × 500 nm^2^; **b**, **d** 190 × 230 nm^2^; scale bars represent a length of 50 nm; top-right insets: 15 × 15 nm^2^), and LEED patterns of the SrTiO_3_(110)–(4 × 1) (**a**, **c**) and –(2 × 5) (**b**, **d**) surfaces. The as-prepared (**a**, **b**) and O_2_-annealed (**c**, **d**) samples appear similar; the slightly different contrast observed in the high-resolution STM images of **b** and **d** are related to variations of the tip termination

Representative scanning tunneling microscopy (STM) images and low-energy electron diffraction (LEED) patterns from these two surfaces are shown in Fig. 2. The six- and ten-membered rings of TiO_4_ on the (4 × 1) surface give rise to rows running along the $\left[ {1\bar 10} \right]$ direction, separated by darker trenches along [001], as seen in the high-resolution STM images. Bright, isolated features (light-blue arrows in the top-right inset of Fig. 2a) have been identified as single Sr adatoms, preferentially occupying a surface domain boundary^34^. In wide-area STM images, (4 × 1)-reconstructed SrTiO_3_(110) surfaces exhibit large (20–300 nm), atomically flat terraces, separated by steps with single (275 pm) or multiple unit-cell heights, preferentially running along low-index directions (see main panel of Fig. 2a). On the (2 × 5) surface, Sr atoms close to the TiO_5_ units are usually predominantly visible in high-resolution STM (indicated by the light-blue arrows in Fig. 2b). These Sr atoms are imaged as protrusions centered on shallow dark trenches, while the surface TiO_6_ octahedra are responsible for the bright appearance of the stripes extending along the [001] direction^31^. The inset shows the characteristic LEED pattern with (2 × 5) periodicity. In large-area STM images, the (2 × 5)-reconstructed SrTiO_3_(110) surface shows flat terraces with a morphology similar to the (4 × 1) reconstruction.

After annealing these samples in oxygen atmosphere prior to the ^18^O exchange experiment (450 °C, 0.1 mbar O_2_, for 5 h), the atomic-scale structure appears essentially unchanged for both surfaces, seen from comparing Fig. 2a, b with Fig. 2c, d. The (4 × 1)-reconstructed surface (Fig. 2c) retains the atomically flat, large-scale morphology of the pristine sample. On the SrTiO_3_(110)-(2 × 5) surface (Fig. 2d), the morphology is also largely preserved, along with a slight increase of the fraction covered by rectangular islands. It is known that even small changes in the surface Ti/Sr ratio would switch the surface to another reconstruction^35^, thus we can exclude any cation segregation. These surface structures are stable upon annealing under the same conditions for a total of 20 h, see Supplementary Fig. 2.

We also prepared a “surface bi-crystal,” i.e., a sample with zones of (4 × 1) and (2 × 5) surface structures. The bulk of the material has thus exactly the same sample history and composition, only the surfaces are different. Sample work functions were determined by measuring the cutoff of secondary electrons (excited by X-rays); the values are 4.470(3) eV and 4.051(10) eV for the (4 × 1) and (2 × 5) reconstructions, respectively (data shown in Supplementary Fig. 5). By comparing the core-level energies in X-ray photoelectron spectra (XPS) of the two zones on the surface bi-crystal (see Supplementary Fig. 6), no difference in band bending was found, indicating that any surface potential difference is negligible within the measurement uncertainty (±0.03 eV).

### ^18^O_2_ exchange kinetics from ion spectroscopies

**Fig. 3:**
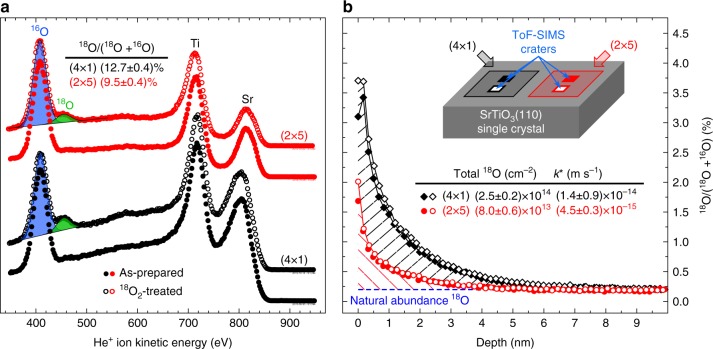
Oxygen-isotope-sensitive ion spectroscopy measurements performed on the SrTiO_3_(110)-reconstructed surfaces. **a** LEIS spectra (He^+^ with 1000 eV primary energy) measured on (4 × 1)- (black), and (2 × 5)-reconstructed (red) SrTiO_3_(110) surfaces, respectively. Results from as-prepared and ^18^O-exchanged samples are displayed with full and open symbols, respectively. Each spectrum is normalized to the total O signal. The uncertainty (standard error) on the fractional ^18^O signals reported in the table is 0.4% for both surfaces. **b** ToF-SIMS ^18^O isotope exchange depth profiles measured on (4 × 1)- (black), and (2 × 5)-reconstructed (red) SrTiO_3_(110) surfaces of the same crystal. Here, open and full symbols correspond to two separate measurements on different spots on each half of the bi-crystal

^18^O exchange was conducted on monophase samples and on the surface bi-crystal sample. The resulting tracer incorporation was evaluated with secondary ion mass spectrometry (SIMS) and low-energy He^+^ ion scattering (LEIS), see Fig. 3. The LEIS spectra were acquired on the monophase samples in ultra-high vacuum (UHV) right after the ^18^O treatment (Fig. 3a). Figure 3b shows representative SIMS depth profiles for the ^18^O isotope on the different zones of the surface bi-crystal; similar results were achieved for the monophase samples (Supplementary Fig. 1). In each case, the (4 × 1)-reconstructed surface incorporates significantly more ^18^O during the exchange process than the (2 × 5)-reconstructed surface.

Quantification of tracer incorporation at the surface is commonly assessed through the determination of the exchange coefficient k*. This is usually accomplished by fitting the measured tracer fraction profile to analytical or numerical solutions of Fick’s law of diffusion. However, this approach is not applicable in our case, since the profiles in Fig. 3b are extremely shallow due to virtual absence of oxygen vacancies in the bulk of donor-doped SrTiO_3_ (Supplementary Note 6), and are dominated by SIMS-related broadening effects (see Supplementary Note 3 and Supplementary Fig. 4). The total amount of incorporated ^18^O, however, can still be correctly determined by integration of the ^18^O profiles (see Fig. 3b). From this total amount of exchanged ^18^O, an oxygen exchange rate coefficient k* can be obtained (see Methods and Supplementary Eqs. (1–5)). With exchange times of 1 h and 4 h, we find k* to be 4.5–6.0 × 10^−15^ m s^−1^ for the (2 × 5)-reconstructed surface, and approximately three times as much (1.4–1.8 × 10^−14^ m s^−1^) for the (4 × 1) (Fig. 3 and Supplementary Fig. 3). Moreover, the ratio of effective surface exchange constants k*_(4×1)_/k*_(2×5)_ amounts to 3.1 ± 0.3 and 3.1 ± 0.6 for the 1 h and 4 h annealing periods, respectively, implying that the incorporation via the surface remains constant over time, at least over several hours. We can thereby exclude that either saturation effects, or different transport in the near-surface regions are responsible for the observed differences between the two reconstructions. Consequently, only the different activity of the respective surface structures is responsible for the threefold larger ^18^O incorporation on the (4 × 1).

It has to be noted that, with respect to LEIS (Fig. 3b), SIMS (Fig. 3a) measures a larger difference in incorporated oxygen between (4 × 1) and (2 × 5). The reason lies in the different probing depths of the two techniques: While SIMS probes the total amount of tracer ions incorporated in the samples, LEIS is strictly surface sensitive, and therefore probes the ^18^O density in the topmost surface layer only. This surface ^18^O density is obtained by scaling the LEIS-measured tracer concentrations with the corresponding density of oxygen atoms in the topmost layer [8.81 × 10^14^ cm^−2^ and 1.04 × 10^15^ cm^−2^ for (2 × 5) and (4 × 1), respectively], and amounts to (8.4 ± 0.4) × 10^13^ cm^−2^ for (2 × 5) and to (1.33 ± 0.04) × 10^14^ cm^−2^ for (4 × 1). The ^18^O density on the (2 × 5) surface nicely agrees with the total SIMS signal [(8.0 ± 0.6) × 10^13^ cm^−2^], whereas on (4 × 1) it amounts to half of the total SIMS counts [(2.5 ± 0.2) × 10^14^ cm^−2^]. This strongly suggests that oxygen exchange is confined to the topmost surface layer on the (2 × 5), while on the (4 × 1) a few atomic layers are involved. This argument is further strengthened by DFT calculations (see below), which show that oxygen vacancies are more prevalent at the very surface on the (2 × 5), while subsurface sites are favored on the (4 × 1). Moreover, the slight broadening of the SIMS profile found for (4 × 1) after long exchange time (see Supplementary Note 3) supports this conclusion.

### O_2_ reactivity from DFT calculations

The availability of well-supported atomic surface models^31,34,35^ (Fig. 1) and the fact that these structures are not affected by annealing in ^18^O_2_ (Fig. 2) allow us to directly relate the experimental results to first-principles calculations. We first evaluated the formation energies for O vacancies at various positions within the reconstruction layer, both on the top of the surface and closer to the interface with the underlying SrTiO_3_ bulk lattice (Supplementary Fig. 7 and Supplementary Table 1). The formation of the lowest-energy V_O_ on the (2 × 5) surface layer is more favorable by 2.2 eV than on the (4 × 1) surface. Thus, ease of surface V_O_ formation is clearly not a decisive factor in the ^18^O exchange.

**Fig. 4:**
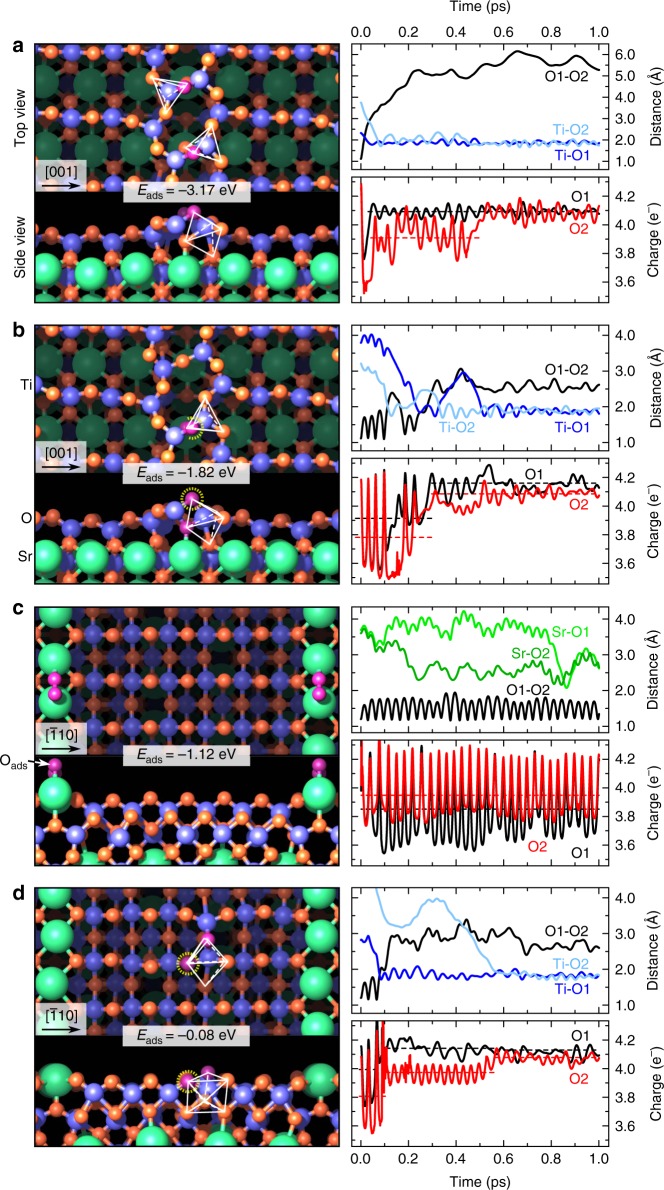
O_2_ adsorption structures and O_2_ dynamics calculated by FPMD-DFT. Lowest-energy structural models and time evolution (first 1.0 ps; for later time the results remain unchanged) of selected ionic distances and charge populations during the adsorption of one O_2_ on **a** the (*n* × 1) and **c** the (2 × *n*) surfaces without a vacancy, and on **b** the (*n* × 1) and **d** the (2 × *n*) surfaces with one V_O_ (the O atom filling the V_O_ is highlighted with a dashed yellow circle). Adsorption energies are indicated, and take into account also the V_O_ formation energy. Green, red, and blue spheres indicate Sr, O, and Ti atoms, respectively. The intact, adsorbed O_2_ molecule and the O atoms resulting from its dissociation are depicted in purple. Five- (**a**, **b**) and sevenfold (**d**) coordination polyhedra of Ti are outlined in white. O1 and O2 refer to the oxygen atoms belonging to the O_2_ molecule, while Ti–O1 (Ti–O2) is the distance between O1 (O2) and the closest Ti atom in the structure

Next, we modeled with first-principles molecular dynamics (FPMD) the interaction of O_2_ considering a vacancy-free surface and a defective one with a single V_O_ for each of the two reconstructions. The adsorption structures with the lowest energy at the end of the FPMD runs are displayed in Fig. 4, along with the corresponding relative adsorption energies *E*_ads_. These structures are thermodynamically stable, as proven by the phase diagram shown in Supplementary Figs. 11 and 12. Additional (less stable) configurations are shown in Supplementary Fig. 8 and Supplementary Table 2. The kinetics of the entire process of oxygen incorporation depends on the adsorption energies as well as the unit reaction energy barriers. The rate of incorporation of oxygen from surface to subsurface is affected by the concentration of adsorbed oxygen at the surface. That term, the source of oxygen incorporation, is determined by adsorption energies. For the vacancy-free surfaces, O_2_ dissociative adsorption/incorporation is considerably more favorable in the (4 × 1) structure. Even without a V_O_, O_2_ easily dissociates and incorporates into the (4 × 1) surface at both the six-member and the ten-member rings of TiO_4_ tetrahedra, with an energy gain of −3.17 eV and −2.94 eV, respectively. On the vacancy-free (2 × 5) surface, in contrast, O_2_ does not dissociate. It stays anchored on top of the Sr rows (see Fig. 4c). O_2_ adsorption and dissociation is also energetically highly unfavorable on the flat TiO_6_ area, resulting in a positive adsorption energy (see Supplementary Fig. 8). If V_O_s are present in the (2 × 5) structure, they play the role of an active center for O_2_ dissociation (see Fig. 4d): one O fills the V_O_ site, whereas the second one hops into a nearby bridge position between two surface Ti atoms. We note here that it is important to consider and compare these adsorption energies in the kinetics of oxygen incorporation.

These results can be rationalized by inspecting the time evolution of the relevant ionic distances and the O_2_ valence charge during the dissociation of the O_2_ molecule in the (4 × 1) and (2 × 5) surfaces without and with vacancy^36^, see Fig. 4. In the (4 × 1) phase, the O1–O2 bond of the O_2_ molecule breaks rapidly during the initial 0.3 ps, and the O1/O2 valence charges increase by about 0.3 electrons as a result of charge transfer from the surface Ti atoms. The O_2_ dissociation mechanism in the vacancy-free (4 × 1) surface is different compared to the defective surface. Without V_O_ the dissociated O atoms bind to surface Ti atoms located on opposite sides of the six-member ring, as confirmed by the increasing O1–O2 distance and the decrease of the Ti–O distances. Instead, on the defective surface, one O atom remains at the surface filling the V_O_ site and re-establishing the Ti–O bond, while the second one is incorporated in the subsurface, increasing the coordination of the Ti atoms from TiO_4_ to TiO_5_. The overall energy gain, −1.82 eV, is reduced by about 1.3 eV with respect to the vacancy-free surface due to the large energy cost to form V_O_s (see Supplementary Table 1). A similar dissociation mechanism is at play on the defective (2 × 5) surface (Fig. 4d) but the final energy gain is much smaller—by 1.74 eV—compared to (4 × 1). Conversely, on the vacancy-free (2 × 5) surface, the O1–O2 bond does not break (the bond length oscillates between 1.2 Å and 1.6 Å), and the O_2_ molecule binds weakly to the underlying Sr atoms.

We have also computed the activation energy for the O_2_ dissociation process on both surfaces with nudged elastic band (NEB) calculations. Since O_2_ dissociation is endothermic on the vacancy free (2 × 5) (Supplementary Fig. 8), we have only considered the vacancy-free (4 × 1) and both the (4 × 1) and (2 × 5) surfaces with one V_O_. The results, collected in Supplementary Fig. 9, deliver low transition barriers: 0.3 eV for the clean and defective (4 × 1), and 0.1 eV for the defective (2 × 5) reconstruction.

**Fig. 5:**
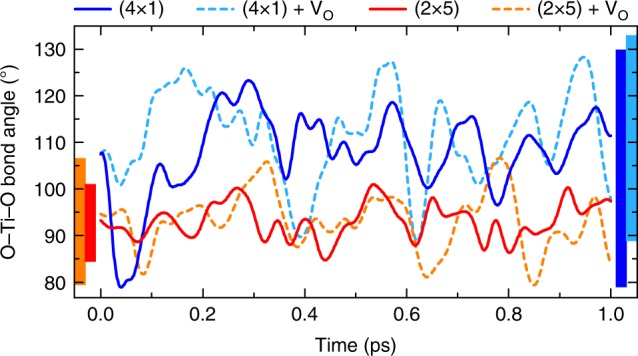
Dynamical reorganization of the TiO_4_ and TiO_6_ polyhedra. Time evolution of the average O−Ti−O angle in the (4 × 1) and (2 × 5) surfaces with and without oxygen vacancy. Trajectories are shown during the first 1.0 ps, but qualitatively similar results are obtained for the whole time range considered in the calculation (10 ps). Vertical bars indicate the maximum amplitude of the oscillations during the whole simulation period

We attribute the different reactivity of the (4 × 1) and (2 × 5) surfaces to the different degree of structural flexibility of the TiO_4_ and TiO_6_ surface polyhedra, manifested by the dynamical structural reorganization (rotations and distortions associated with phonon softening, see Supplementary Note 10 and ref. ^37^) of the TiO_4_ and TiO_6_ units during the adsorption process. The time evolution of the average O–Ti–O angle of surface polyhedra in the (4 × 1) and (2 × 5) surfaces, displayed in Fig. 5, shows that the under-coordinated TiO_4_ polyhedra undergo much larger oscillations compared to the TiO_6_ units. The ease of performing such structural distortions ultimately provides the (4 × 1) surface with the structural flexibility to host external adsorbates such as O_2_, and off-stoichiometry facilitates charge transfer from the under-coordinated Ti atoms to the adsorbed oxygen. In contrast, stoichiometric TiO_6_ octahedra are structurally rigid and chemically saturated, and active adsorption and migration is reduced or suppressed, unless oxygen vacancies that reduce the TiO_6_ coordination are formed. In other words, such dynamic reorganization performs similarly to a mobile oxygen vacancy at the surface, and represents an alternative way to promote the oxygen incorporation reaction.

## Discussion

Our experimental and computational results are at odds with the commonly accepted models for oxygen exchange mentioned above, i.e., availability and mobility of V_O_s^6–10^ and the electronic effects that facilitate electron transfer^11–14^. Our experimental and theoretical methods allow us to independently assess these factors: their values suggest a higher reactivity of the (2 × 5) structure, the opposite of what is observed in the ^18^O experiments.

From our DFT calculations (Supplementary Fig. 7 and Supplementary Table 1), the (2 × 5) surface should accommodate a larger concentration of oxygen vacancies at the surface compared to (4 × 1). The preferential vacancy formation sites on the (4 × 1) system are, in fact, sub-surface^38^. Therefore, the vacancy mechanism cannot explain the higher reactivity of the (4 × 1) compared to the (2 × 5) surface. When it comes to the ease of electron transfer, the surface work function is a good measure to correlate to reactivity^11–14^. The work function measured by XPS and calculated by DFT is lower by 0.42 eV and 0.7 eV, respectively, for the (2 × 5) compared to the (4 × 1) surface. The core-level binding energies measured on the (4 × 1) and the (2 × 5) surfaces are the same (Supplementary Fig. 6). Therefore, the surface potential or band bending (if it exists) is the same on these two surfaces, and the vacuum level on the (2 × 5) surface should be lower. In addition, the (4 × 1) surface has a larger band gap than the (2 × 5)^38^. Therefore, electron transfer to O_2_ molecules should be easier at the (2 × 5) surface. Again, this cannot explain why (4 × 1) is more reactive. In addition, from our STM images it is clear that the step density, which can significantly enhance reactivity^39^, is comparable on both surfaces, or slightly higher at the (2 × 5) surface after oxygen exchange treatment (Fig. 2). Thus, step density cannot be the reason for the higher reactivity of the (4 × 1) either. Consequently, none of these traditionally considered models related to vacancies, electronic structure, or step edges for controlling the reactivity of oxide surfaces are able to explain the difference found between the reactivities of the (4 × 1)- and (2 × 5)-reconstructed surfaces on SrTiO_3_(110).

Our computational results reveal that oxygen incorporation on SrTiO_3_(110) is affected directly via the atomic structure itself. The lower coordination of Ti by O atoms on the (4 × 1) accommodates the adsorbed/dissociated oxygen by increasing the coordination of Ti, either at the top surface or by incorporating oxygen atoms to the first subsurface layer. In other words, the enhanced reactivity of the (4 × 1) toward O_2_ dissociative adsorption and incorporation is a result of the increased degree of structural and chemical flexibility of the under-coordinated TiO_4_ units as compared to the almost fully coordinated and rigid TiO_5_ and TiO_6_ units in the (2 × 5). The rigid TiO_6_ polyhedra in the (2 × 5) surface cannot accommodate more oxygen unless surface V_O_s assist the reaction.

Thus, we suggest that two parallel oxygen exchange mechanisms are present on our SrTiO_3_ surfaces: a structurally mediated mechanism, dominating the exchange rate on the (4 × 1) surface, and a vacancy-mediated one, enabling exchange also on the (2 × 5) surface.

Existence of a vacancy-mediated oxygen exchange is often assumed in the literature, and the oxygen exchange rate is frequently compared with the (bulk) V_O_ concentration^6^. A direct and proportional dependence of the k* on the oxygen vacancy concentration in the bulk was shown for some perovskite oxides^6^. k* divided by the fraction of oxygen sites occupied by V_O_s, denoted here as k*_V_, amounts to 10^−6^ m s^−1^ and 10^−5^ m s^−1^ for LSCF and BSCF, respectively, at 800 °C in air^6^, and to 10^−9^–10^−8^ m s^−1^ for LSC at 450 °C and 200 mbar O_2_^40^. On (2 × 5)-reconstructed surfaces of our 0.5 wt.% Nb-doped SrTiO_3_(110), k*_V_ is at least on the order of 10^−4^ m s^−1^ at 450 °C and 0.1 mbar oxygen (see Supplementary Note 6 for estimates of the vacancy concentration in our samples). Intriguingly, this value is comparable to that on LSCF and BSCF, and several orders of magnitude higher than the values reported for LSC. A possible factor contributing to this large discrepancy is the completely clean and stable atomic structures that are retained without any segregation of cations, impurities, or secondary phases on our samples. The often-found surface segregation and phase precipitation of SrO_*x*_ or impurities on the aliovalently doped systems hinder their surface reactivity by blocking the electron and oxygen transfer reactions^30,41,42^. Indeed, a substantially enhanced k* was found for a clean LSC surface upon removal of the SrO_*x*_ termination layer^43^. Presumably, for vacancy-rich perovskite oxides the full potential of oxygen activity has not been exploited yet. These findings further demonstrate that establishing physics-based relations of the very surface state to the reaction kinetics is needed for a mechanistic understanding, rather than relying only on bulk non-stoichiometry of oxygen as a descriptor.

In summary, we have realized a comprehensive and gap-bridging approach between model surfaces with well-defined atomic structure and macroscopic kinetic measurements of reaction rates, and revealed a dependence of reactivity on the precise surface atomic structure on a perovskite oxide. The oxygen exchange kinetics is three times faster on the (4 × 1)-reconstructed surface of SrTiO_3_(110) than on the (2 × 5) one. Neither availability of surface vacancies nor the work function or band bending can explain this enhanced reactivity of the (4 × 1) surface. We find that the surface atomic structure itself has the dominant role by assisting the oxygen adsorption and dissociation on the (4 × 1) surface, owing to a high degree of dynamical flexibility of the under-coordinated surface Ti atoms. This directly demonstrates and explains the influence of reconstructions on the reactivity of a perovskite oxide. Reconstructions are prevalent at the surfaces of any complex oxide^44^, and oxides with other transition metal cations such as Fe, Mn, and Co can as well form different polyhedral coordinations^45,46^. Accordingly, the higher flexibility of unsaturated metal–oxygen polyhedra may play a role in affecting the ease of oxygen incorporation on a broader range of perovskite oxide surfaces. Most importantly, the gap-bridging approach that we demonstrated here should motivate more research in resolving the surface atomic structure and relating that to reactivity on a wider range of perovskite oxides.

## Methods

### Sample preparation

The surfaces of one-side polished, Nb-doped (0.5 wt.%) SrTiO_3_(110) single crystals (5 × 5 mm^2^, Crystec GmbH) were prepared in an ultra-high vacuum (UHV) system^47^. The latter is composed of three in situ interconnected vessels: The preparation chamber is used for Ar-ion sputtering, electron-beam annealing, and contains molecular beam epitaxy (MBE) facilities. The analysis chamber houses a low-energy electron diffraction (LEED) setup, a hemispherical analyzer for X-ray photoelectron (XPS) and low-energy ion scattering (LEIS; 1 keV He^+^; scattering angle 132°) spectroscopies, and a variable-temperature scanning tunneling microscope (STM). The oxygen exchange experiments were performed in the third vessel (pulsed laser deposition chamber), allowing for infrared-laser annealing of samples at elevated oxygen pressures (up to 200 mbar).

The SrTiO_3_(110) crystals were cleaned in the preparation chamber by repeated cycles of sputtering (1 keV, 5 μA, 10 min) and annealing in O_2_ gas (1000 °C, 3 × 10^−6^ mbar, 1 h), to remove contamination. The surface of each sample was then prepared to exhibit predominantly a (4 × 1) or a (2 × 5) structure. The (4 × 1) structure was obtained by deposition of sub-monolayer amounts of Sr or Ti at room temperature, and subsequent annealing in an O_2_ background (1000 °C, 3 × 10^−6^ mbar, 30 min)^35^. Particular care was taken in obtaining a uniform structure over the whole sample surface, by deposition of small amounts of metals while partly shadowing the sample with a dedicated shutter. The (2 × 5)-reconstructed surfaces were obtained by reactively depositing (5 × 10^−6^ mbar O_2_) 0.8 Å Ti at 600 °C on SrTiO_3_(110)-(4 × 1). Two sets of samples were prepared: either single-phase [uniformly (4 × 1)- or (2 × 5)-reconstructed on a given crystal], to allow for STM characterization, or with both the (4 × 1) and (2 × 5) co-existing on the same crystal surface, each occupying ~5 × 2.5 mm^2^. In the latter case, half of the sample surface was shadowed during reactive deposition of Ti to induce the (2 × 5) surface after the (4 × 1) surface was formed. We refer to the latter as a “surface bi-crystal.” The results obtained with the two sets of samples were checked for consistency by comparing the LEED patterns on several spots on the sample at each preparation step, and after the subsequent treatments.

### ^18^O isotope exchange

To ensure the stabilization of a steady-state oxygen vacancy (V_O_) concentration, the as-prepared samples were first annealed in situ in flowing O_2_ gas of natural isotope composition (referred to as ^16^O_2_) for 4 h or 16 h, at 450 °C and 0.1 mbar, with 60 °C min^−1^ heating and cooling ramps. The reason for performing the exchange at a relatively low temperature and oxygen pressure is to minimize the risk of atomic surface structure changes. Furthermore, because of the very low vacancy concentration (as explained above), the O_2_ exchange is expected to be limited to the near-surface region without bulk transport. Prior to the equilibration step, the vessel was continuously flushed with ^16^O_2_ (20 sccm) for 1 h; this ensures reproducible conditions in the vacuum chamber. The pumping speed was regulated to stabilize a pressure of 1 mbar. Finally, each sample was annealed in isotopically labeled oxygen (97.1% ^18^O_2_, referred to as ^18^O_2_ for brevity) for 1 h or 4 h, at 450 °C and 0.1 mbar (static) with heating and cooling ramps of 60 °C min^−1^ and 120 °C min^−1^, respectively. Similar to the ^16^O_2_ equilibration step, the chamber was pre-conditioned in ^18^O_2_ at 0.5 mbar static pressure for 30 min prior to each experiment. After the ^18^O_2_ treatments, samples were analyzed with LEED, XPS (both monophase and surface bi-crystal samples), STM, and LEIS (monophase samples only) in situ, i.e., in the UHV apparatus, and then transported to the time-of-flight–secondary-ion mass spectrometry (ToF-SIMS) setup.

### Depth profiling and quantification of ^18^O

Oxygen-isotope depth profiles were analyzed via ToF-SIMS on a TOF-SIMS 5 instrument (ION-TOF, Germany). 25 keV Bi_3_^++^ clusters (0.02 pA) were used as primary ions in collimated burst alignment mode optimized for oxygen isotope measurements^18,48^. Negative secondary ions were detected from areas of 100 × 100 µm^2^ using a raster of 512 × 512 measured points, and the secondary ion counts of ^16^O^−^ and ^18^O^−^ were used to determine the isotopic composition *f* = ^18^O/(^18^O + ^16^O). For depth profiling, areas of 400 × 400 µm^2^ were sputtered using Cs^+^ ions, and the depth information was calculated from the sputter coefficients and sputter currents, referenced by measuring the depth of the sputter craters via digital holography microscopy. An electron flood gun (21 eV) was used for charge compensation.

For calculating the average oxygen exchange coefficient k* from the measured fraction of incorporated ^18^O (*f*_m_), the ^18^O fraction in the stainless steel UHV vessel (*f*_out_) was estimated as 0.971, corresponding to the original ^18^O concentration of the tracer-enriched oxygen. The validity of this assumption was tested by exchanging (at 700 °C) ^18^O into a fast-mixed ionic electronic conductor, a 50 nm-thick La_0.6_Sr_0.4_CoO_3_ film grown on SrTiO_3_(001)^40^, under nominally identical gas composition as the exchange experiment performed on the SrTiO_3_(110) crystals in the same UHV setup. Since *f*_out_ is much larger than all tracer fractions measured in the Nb-doped SrTiO_3_ single crystals, we can approximately assume a constant tracer exchange flux density *j* during the exchange time *t*. Within this assumption, the effective oxygen exchange coefficient can be expressed as (see Supplementary Note 2 for the full derivation)

1 $$\begin{array}{*{20}{c}} {k^ \ast {\mathrm{ = }}\frac{j}{{\left( {f_{{\mathrm{out}}} - f_{{\mathrm{bg}}}} \right)c_O}}{\mathrm{ = }}\frac{{\mathop {\sum }\nolimits_{{\mathrm{meas}}{\mathrm{.}}\,{\mathrm{points}}} \left( {f_m - f_{{\mathrm{bg}}}} \right)d_s/t}}{{\left( {f_{{\mathrm{out}}} - f_{{\mathrm{bg}}}} \right)}}.} \end{array}$$

In Eq. (1), *f*_bg_ denotes the background tracer fraction (approximated by the natural abundance *f*_bg_ = 0.00205), *c*_O_ is the concentration of oxygen sites, and *d*_s_ is the sputter depth per measurement point.

### First-principles calculations

All calculations were carried out using the Vienna ab initio simulation package (VASP)^49,50^ in the framework of density functional theory (DFT) within the generalized gradient correction approximation of Perdew, Burke and Ernzerhof^51^. The surface reconstructions were modeled with symmetric slabs composed by five layers plus the reconstructed surfaces, separated by ~12 Å of vacuum. We used the same (4 × 4) two-dimensional base unit cell for both types of SrTiO_3_(110) reconstructions, effectively modeling (4 × 2) and (2 × 4) reconstructions. (4 × 2) and (4 × 1) belong to the same (*n* × 1) structure family, and (2 × 4) and (2 × 5) belong to the same (2 × *n*) surface structure family. For consistency with the experimental description, we refer to the slabs representing the (*n* × 1) overlayer as (4 × 1), and the (2 × *n*) overlayer as (2 × 5) in the text. The surface geometry was optimized by keeping the central three layers fixed to the corresponding bulk positions and relaxing the remaining atomic positions until the forces on each atom were less than 0.02 eV Å^−1^. For the Brillouin-zone integration, we have adopted a 2 × 2 × 1 **k**-point grid, corresponding to an 8 × 8 × 1 mesh for the 1 × 1 cell, and used a standard energy cutoff of about 300 eV for the plane-wave expansion. The formation energy of one oxygen vacancy formed on both sides of the symmetric slab was computed using the standard relation

2 $$\begin{array}{*{20}{c}} {E_{\mathrm{f}}\left( {{\mathrm{V}}_{\mathrm{O}}} \right) = \frac{1}{2}\left[ {E_{{\mathrm{tot}}}\left( {2{\mathrm{V}}_{\mathrm{O}}} \right) + E_{{\mathrm{O}}_2}^{{\mathrm{mol}}} - E_{{\mathrm{tot}}}\left( {{\mathrm{0V}}_{\mathrm{O}}} \right)} \right],} \end{array}$$

where $E_{{\mathrm{O}}_2}^{\rm{mol}}$ is the DFT energy of an isolated oxygen molecule, whereas *E*_tot_(2V_O_) and *E*_tot_(0V_O_) represent the DFT total energies of the slabs with and without oxygen vacancies, respectively.

The interaction between the surfaces and oxygen atoms was modeled by studying the adsorption, dissociation, and dynamics of one O_2_ molecule by means of first-principles molecular dynamics (FPMD) and the climbing image nudged elastic band (CI-NEB)^52^. To reduce the computational costs, the FPMD and CI-NEB calculations were performed using asymmetric slabs with a reduced energy cutoff (250 eV) and a smaller **k**-mesh (1 × 1). The FPMD calculations were performed at the simulating temperature of 700 K for 5–10 ps using a canonical ensemble and the Nosé thermostat algorithm^53^. For the estimation of energy barriers within the CI-NEB method, five images were constructed, and the convergence criteria for the forces was reduced to 0.05 eV Å^−1^.

Relative adsorption energies *E*_ads_ per O_2_ molecule, as given in the text, are defined as $E_{{\mathrm{ads}}} = E_{{\mathrm{slab}}}-E_{{\mathrm{clean}}}-E_{{\mathrm{O}}_2}^{{\mathrm{mol}}} + xE_{{\mathrm{V}}_{\mathrm{OS}}}$, where *E*_slab_ and *E*_clean_ are the *T* = 0 DFT total energies of the full slab containing the O_2_ molecule and the clean surface, respectively. $E_{{\mathrm{V}}_{{\mathrm{OS}}}}$ refers to the oxygen vacancy formation energy at site S, chosen as the energetically least costly V_O3_ and V_O4_ for the (4 × 1) and (2 × 5)-like surfaces, respectively (see Supplementary Note 7), and *x* indicates the number of oxygen vacancies in the slab.

Work functions were computed as the difference between the Fermi energy (valence band maximum) and the vacuum level, extracted from the local potential profile in the direction perpendicular to the slab.

## Electronic supplementary material

Supplementary Information

Description of Additional Supplementary Files

Supplementary Data 1

## Data Availability

The data that support the findings of this study are available from the authors on reasonable request. Source data for Figs. 1 and 4, and Supplementary Figs. 7, 9, and 10 are provided with the paper in Supplementary Data 1.
