# Supplementary material for: Influence of surface atomic structure demonstrated on oxygen incorporation mechanism at a model perovskite oxide
Source: Nat Commun. 2018 Sep 13;9:3710. doi: 10.1038/s41467-018-05685-5 (PMC6137039; doi:10.1038/s41467-018-05685-5)
Supplement: Supplementary file 1 — Supplementary Information [file 41467_2018_5685_MOESM1_ESM.pdf]

Supplementary Information

Influence of Surface Atomic Structure Demonstrated on Oxygen Incorporation Mechanism at a Model Perovskite Oxide

M. Riva *et al.*

Supplementary Figures

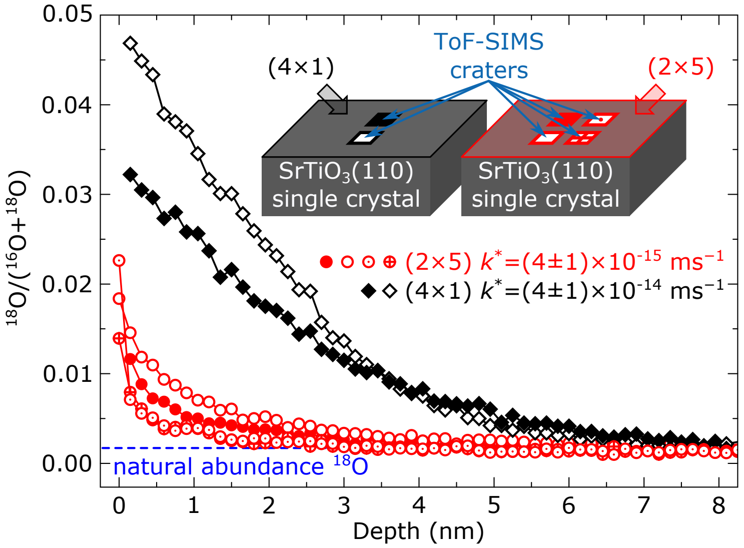

Supplementary Fig. 1 Oxygen isotope exchange depth profiling. ToF-SIMS ^{18}O isotope exchange depth profiles measured on (4 × 1)– (black) and (2 × 5)-reconstructed (red) $SrTiO_3(110)$ surfaces on two different Nb-doped $SrTiO_3(110)$ single crystals. More oxygen is exchanged via the (4 × 1) surface. Values of k^* are calculated assuming a constant oxygen exchange rate during the experiment. See Supplementary Note 1 for details.

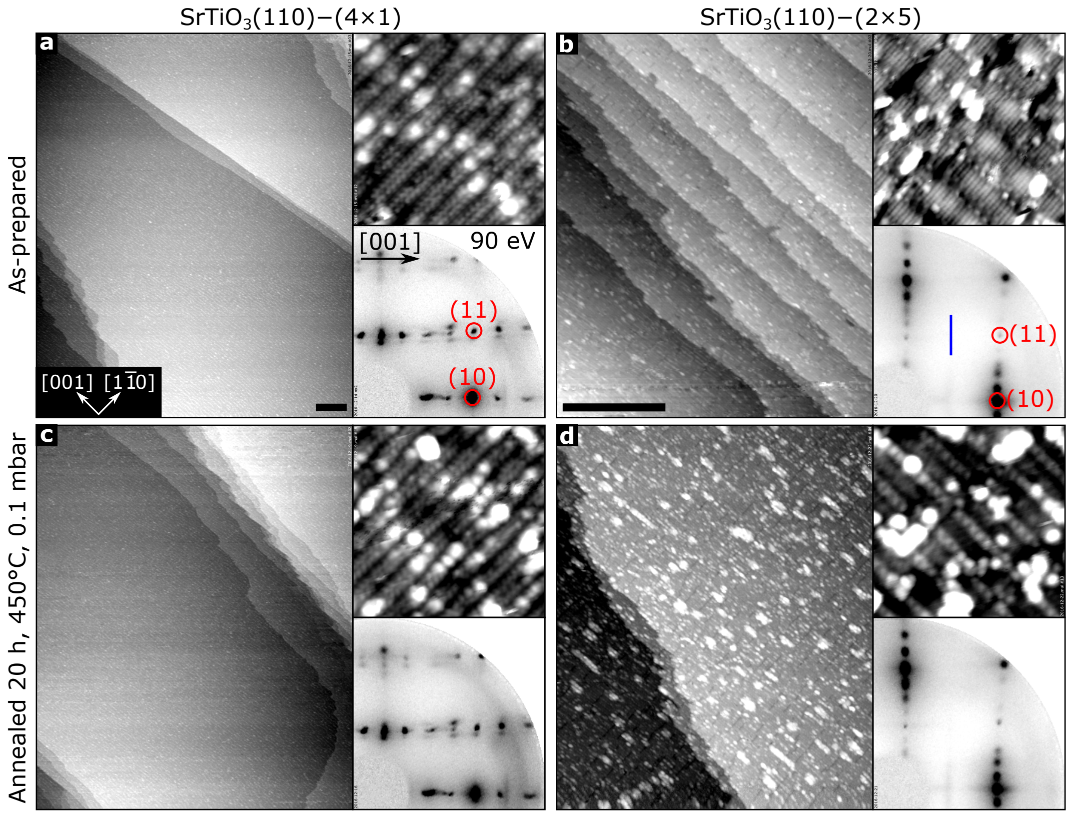

Supplementary Fig. 2 Stability of $SrTiO_3$(110) surface structures upon long annealing. Scanning tunneling microscopy (STM) images (main panels: **a**, **c**, 410 × 500 nm^2; **b**, **d**, 100 × 150 nm^2; scale bars represent a length of 40 nm; top-right insets: 15 × 15 nm^2), and LEED patterns of the $SrTiO_3$(110)-(4 × 1) (**a**, **c**) and -(2 × 5) (**b**, **d**) reconstructed surfaces. The as-prepared (**a**, **b**), and O_2-annealed (20 h at 450°C, 0.1 mbar; **c**, **d**) samples appear similar; the slightly different contrast observed in the high-resolution STM images of panels **b** and **d** are related to variations of the tip termination. See Supplementary Note 2 for details.

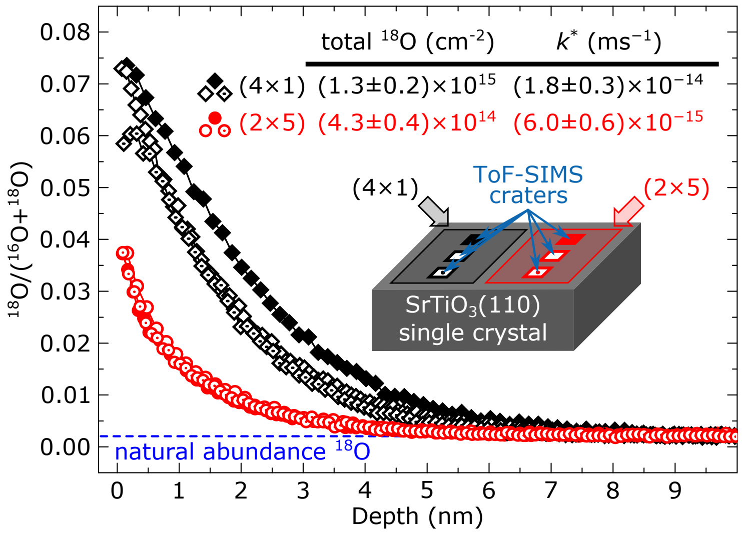

Supplementary Fig. 3 ToF-SIMS ^{18}O isotope depth profiles of a bi-crystal after 4 h exchange period. Black diamonds and red circles correspond to depth profiles acquired on (4 × 1)- and (2 × 5)-reconstructed regions of the $SrTiO_3$(110) surface, respectively. See Supplementary Note 2 for details.

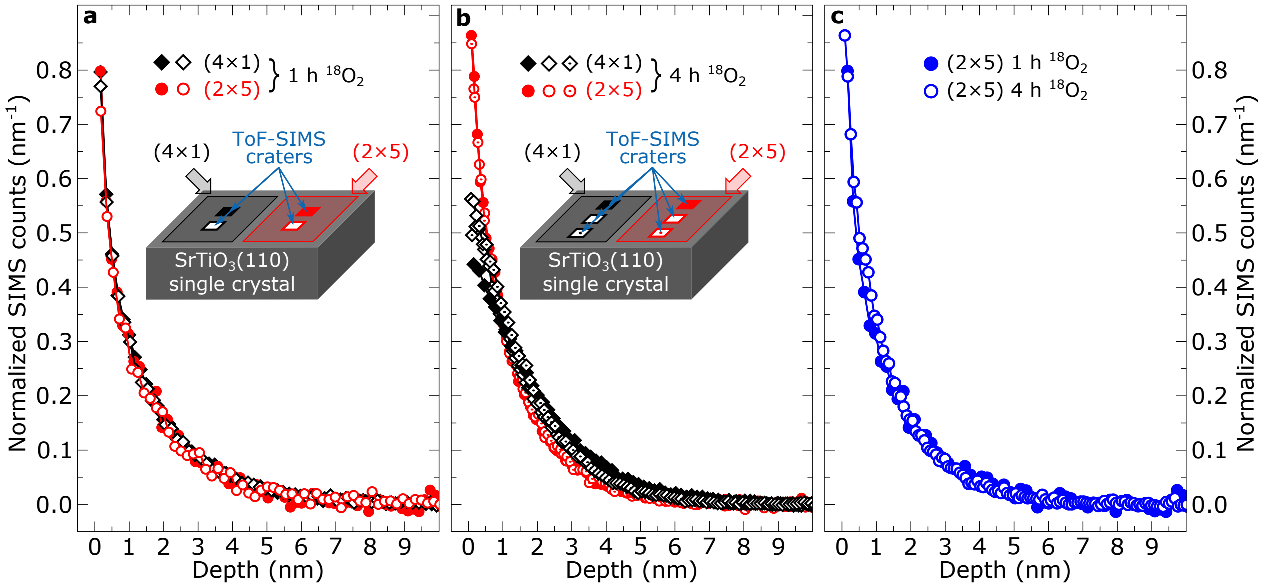

Supplementary Fig. 4 Comparison of ^{18}O depth profile shapes at different exchange durations. SIMS counts measured on the surface bi-crystals after $^{18}O_2$ isotope exchange times of 1 h (**a**) and 4 h (**b**), normalized to the corresponding area under the curve. Black diamonds and red circles represent depth profiles measured on the (4 × 1)- and (2 × 5)-reconstructed areas of the surface bi-crystal, respectively. Here the original SIMS depth profiles (shown in Fig. 3**b** of the main text, and Supplementary Fig. 3) are normalized to the integral counts exceeding the natural abundance. Two of the curves in panels **a** and **b** are compared in panel **c**. It should be noted that, while the normalized depth profiles for the two surface reconstructions measured after 1 h exchange time fall on top of each other (**a**), their respective shapes differ upon longer exchange periods (**b**). Nevertheless, the shape of profiles measured on $SrTiO_3(110)$-(2 × 5) areas (**c**) appears unaffected by the longer annealing periods. See Supplementary Note 3 for details.

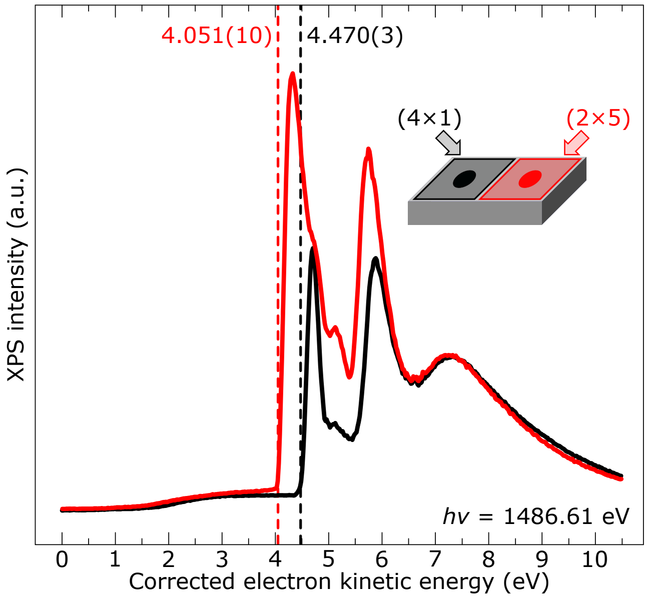

Supplementary Fig. 5 Work function measurement on $SrTiO_3$(110). Secondary-electron emission spectra measured on a surface bi-crystal prepared as described in the main manuscript, showing both (4 × 1) and (2 × 5) reconstructions. Spectra acquired on the two regions are represented in black and red, respectively. The horizontal axis is corrected for the negative bias voltage applied to the sample during measurement (see Supplementary Note 4 for details). Cutoff energies of the secondary electron emission, i.e., work-functions – whose positions are highlighted by vertical dashed lines – were determined by fitting a sigmoidal step function to the main peak in each spectrum. Secondary electrons were collected in normal emission, from an approximately circular region of ~0.5 mm diameter, with 3.5 eV pass energy.

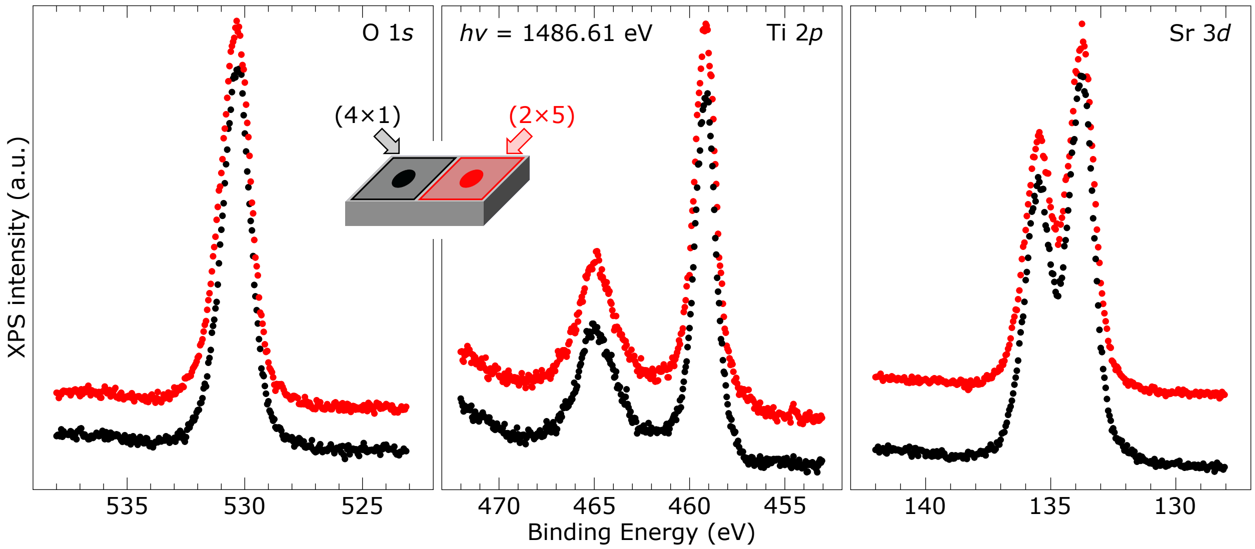

Supplementary Fig. 6 Surface structure-independent band alignment. Core level x-ray photoelectron spectra acquired on the $SrTiO_3(110)$ surface bi-crystal on which the oxygen exchange experiment – described in the main manuscript – was performed. Spectra acquired on the (4 × 1)– and (2 × 5)–reconstructed areas are represented in black, and red, respectively. The spectra were acquired with electron lens settings such that electrons are collected at normal emission from an approximately circular area of ~1.5 mm diameter, and at 10 eV pass energy. The two sets of spectra are vertically offset for clarity. See Supplementary Note 5 for details.

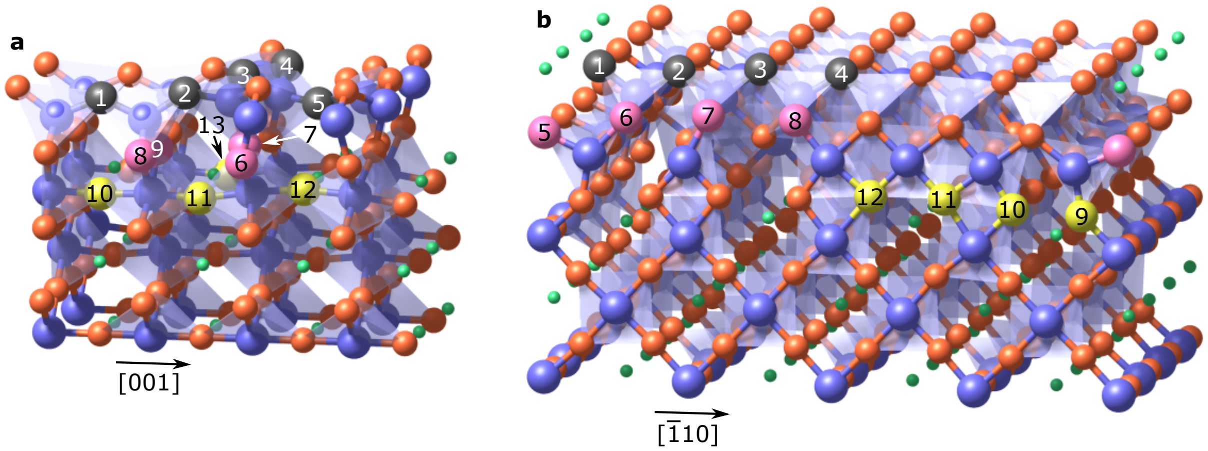

Supplementary Fig. 7 Oxygen vacancies. Structural models for the **a** ($n \times 1$) and **b** ($2 \times n$) reconstructions indicating the positions of the oxygen vacancies considered in our calculations. Green, red and blue spheres indicate Sr, O and Ti atoms, respectively. The oxygen atoms removed to create the vacancies are represented by black (surface, S), pink (sub-surface, S-1) and yellow (interface, I) spheres. All positions are shown here for convenience. Separate calculations were conducted for each individual vacancy. The calculated oxygen vacancy formation energies are listed in Supplementary Table 1. See also Supplementary Note 7 for details.

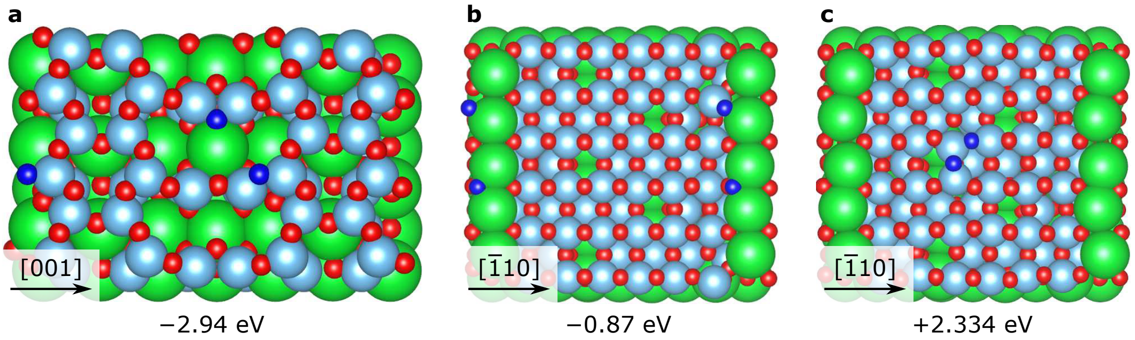

Supplementary Fig. 8 Other configurations for O_2 asdorption and dissociation derived from FPMD. Structural models of additional configurations – as compared to those reported in Fig. 4 of tha main manuscript – for the adsorption and dissociation of one O_2 molecule on the defect-free **a** (4 × 1) and **b** (2 × 5), and on **c** the (2 × 5) with one V_O. The adsorption energies are also indicated. Green, red and light blue spheres indicate surface Sr, O and Ti atoms, respectively. The oxygen atoms originally belonging to the adsorbed O_2 molecule are indicated in dark blue. See Supplementary Note 8 for details.

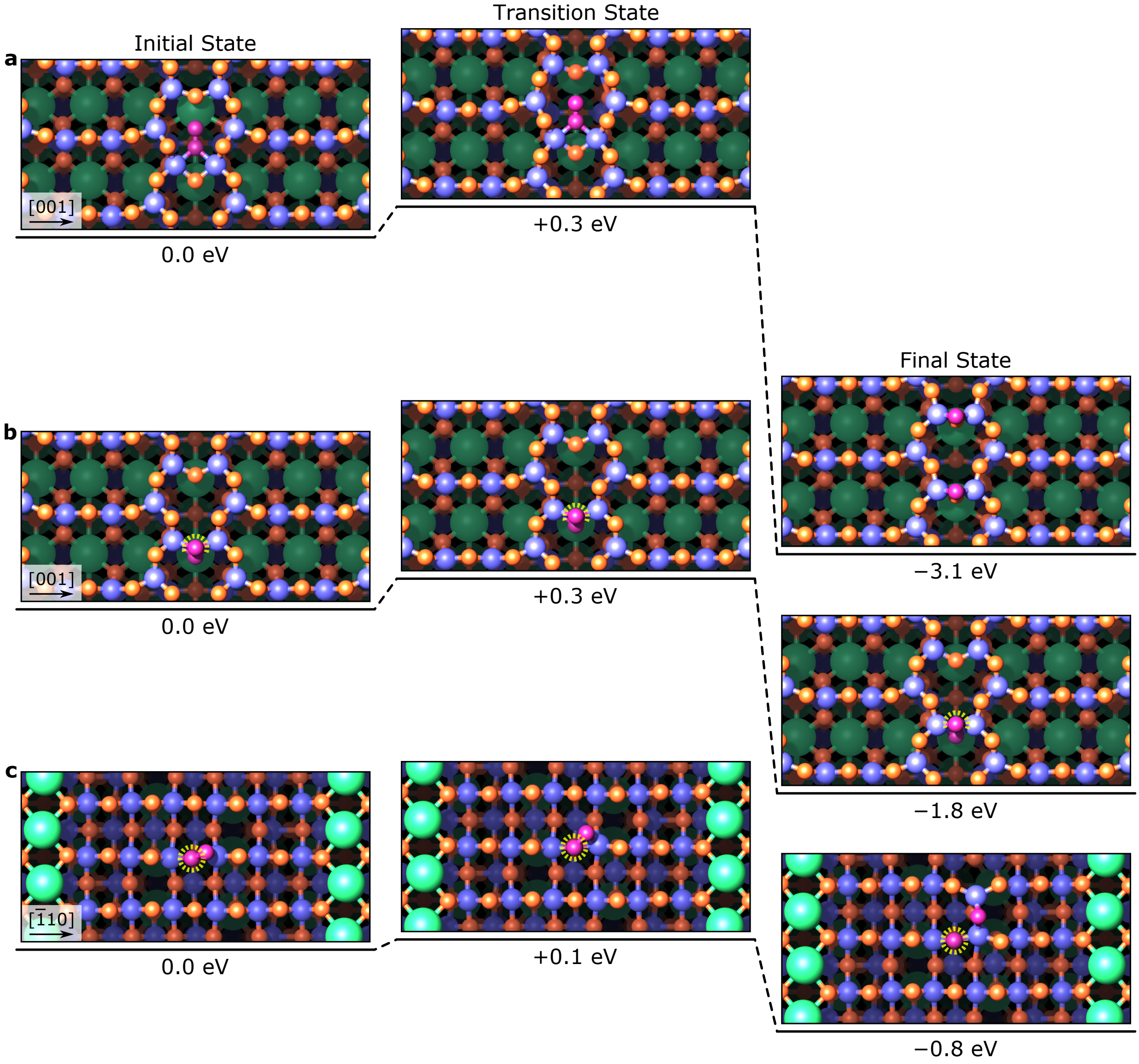

Supplementary Fig. 9 NEB reaction paths describing the adsorption and dissociation of O_2. a Vacancy-free (4 × 1), **b** defective (one V_O) (4 × 1) and **c** defective (one V_O) (2 × 5) surfaces. The initial states correspond to O_2 molecularly adsorbed onto the surface. The O atom filling the initial V_O is highlighted with a dashed yellow circle in panels **b** and **c**. See Supplementary Note 9 for details.

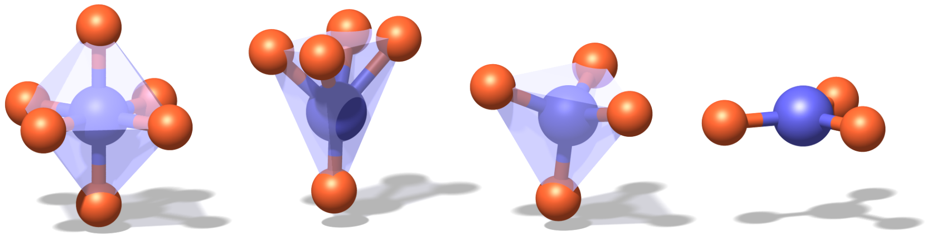

	AO_6	AO_5	AO_4	AO_3
Ti	−11.25	−51.15	−18.07	−27.98
V	−8.45	−30.44	−19.65	−31.40
Cr	−20.68	−34.77	−23.59	−36.93

Supplementary Fig. 10 Flexibility of coordination polyhedra. Force constants (in units of eV $Å^{-2}$) of the lowest rotational mode of the AO_6, AO_5, AO_4, and AO_3 polyhedra for A = Ti, V, Cr. See Supplementary Note 10 for details.

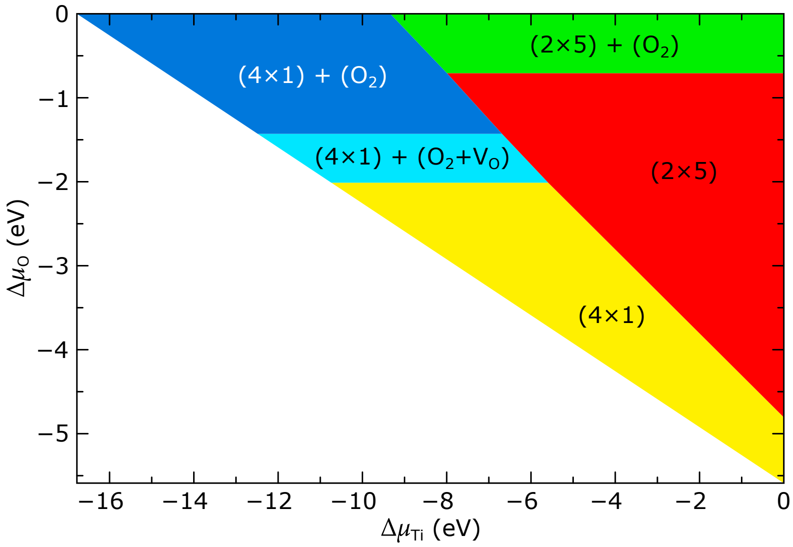

Supplementary Fig. 11 Surface phase diagram, as a function of the O and Ti chemical potentials, of the most stable defect-free and defective phases found by first-principles molecular dynamics for the (4 × 1)/(2 × 5)-reconstructed $SrTiO_3$(110) system. See Supplementary Note 11 for details.

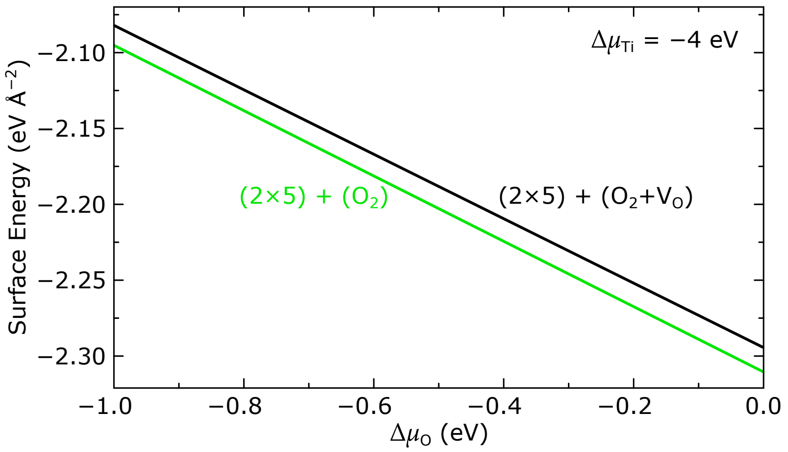

Supplementary Fig. 12 Relative stability of the (2 × 5) + (O_2) and (2 × 5) + (O_2+V_O) structures as a function of the chemical potential of O at a Ti chemical potential of –4 eV (corresponding to the green region in Supplementary Fig. 11). See Supplementary Note 11 for details.

SUPPLEMENTARY TABLES

Supplementary Table 1 DFT-calculated formation energies (in eV) for oxygen vacancies in the (4 × 1) and (2 × 5) reconstructions.

V_O site	(n × 1)	(2 × n)	V_O site	(n × 1)	(2 × n)
V_{O1}	6.43	4.32	V_{O8}	5.59	**4.67 (S-1)**
V_{O2}	5.95	3.87	V_{O9}	5.58	5.10
V_{O3}	**5.60 (S)**	3.82	V_{O10}	**3.56 (I)**	**4.80 (I)**
V_{O4}	5.68	**3.39 (S)**	V_{O11}	3.65	4.88
V_{O5}	5.76	6.14	V_{O12}	4.24	5.20
V_{O6}	5.43	5.48	V_{O13}	3.65	—
V_{O7}	**5.10 (S-1)**	4.75	Bulk	4.40	4.40

Refer to Supplementary Fig. 7 for the assignment of each vacancy site. The most favorable site in each layer (S, S-1, I) is highlighted. See also Supplementary Note 7 for details.

Supplementary Table 2 DFT-calculated O_2 adsorption energies (in eV) for the structures shown in Fig. 4 and Supplementary Fig. 8.

**Structure**		**Adsorption Energy**
(4 × 1), no V_O	Fig. 4a	-3.17
(4 × 1), no V_O	Supplementary Fig. 8a	-2.94
(4 × 1), one V_O	Fig. 4b	-1.82
(2 × 5), no V_O	Fig. 4c	-1.12
(2 × 5), no V_O	Supplementary Fig. 8b	-0.87
(2 × 5), no V_O	Supplementary Fig. 8c	+2.33
(2 × 5), one V_O	Fig. 4d	-0.08

Supplementary Note 1

^{18}O-SIMS on monophase $SrTiO_3$(110)–(4 × 1) and –(2 × 5)

Some SIMS results on Nb-doped $SrTiO_3$(110) single crystals with two different co-existing surface reconstructions ('surface bi-crystal') were presented in the main text of this article. Additional data acquired on similar surface bi-crystals are presented in Supplementary Note 2. In addition to these samples, separate 'monophase' Nb-doped $SrTiO_3$(110) single crystals with only one type of reconstruction, (4 × 1) or (2 × 5), were prepared. The surface structure and morphology were confirmed by STM and LEED. These samples were also subjected to ^{18}O isotope exchange at nominally identical experimental conditions as for the surface bi-crystal described in the main text (i.e., 4 h equilibration in $^{16}O_2$, 1 h annealing in $^{18}O_2$, both at 450°C, 0.1 mbar). On the monophase samples, the oxygen exchange via the (4 × 1) surface is again considerably faster than the exchange via the (2 × 5) surface (Supplementary Fig. 1). The values of the surface exchange coefficient k^* of $(4\pm1)\times10^{-14}$ m s^{-1} and $(4\pm1)\times10^{-15}$ m s^{-1} agree well with those obtained on the surface bi-crystal, i.e., $(1.4 \pm 0.9) \times 10^{-14}$ m s^{-1} and $(4.5 \pm 0.3) \times 10^{-15}$ m s^{-1} for (4 × 1) and (2 × 5), respectively. As slight variations of the annealing conditions (temperature, gas atmosphere) or the individual single crystals could cause some scatter between monophase samples, we put more emphasis on the results obtained on the surface bi-crystals.

Supplementary Note 2

Effect of exchange duration, and derivation of k^* from SIMS data

In order to evaluate the influence of the total exchange time on the amount of incorporated oxygen, and exclude possible saturation effects, a second surface bi-crystal was prepared with a procedure similar to the one described in the main manuscript. Subsequently, this surface bi-crystal was annealed in $^{16}O_2$ (16 h, 450°C, 0.1 mbar), and $^{18}O_2$ (4 h, 450°C, 0.1 mbar), following a procedure analogous to the one described in the main text. In this case, however, the preparation of the surface bi-crystal – first with a uniform (4 × 1) surface structure and then with the additional (2 × 5) reconstruction – was carried out by pulsed laser deposition (248 nm-KrF excimer laser; spot-size: 0.7 × 1.7 mm^2; fluence: 2.5 J cm^{-2}; repetition frequency: 1 Hz; target-substrate distance 55 mm)[1], from a single-crystalline rutile TiO_2 target (pre-ablated before each deposition at the growth parameters), in O_2 background (5×10^{-6} mbar). A total of 27 laser pulses were deposited at 600°C onto

the uniform (4 × 1)–reconstructed $SrTiO_3(110)$ surface – while shading half of the sample with a dedicated mask – to obtain a well-ordered $SrTiO_3(110)$–(2 × 5) surface structure. Comparison of Supplementary Fig. 2a,b and Fig. 2a,b (main manuscript) shows that this PLD-based preparation method yields analogous results for both the $SrTiO_3(110)$–(4 × 1) and –(2 × 5) surface structures. In particular, similar morphologies (main panels of Fig. 2a,b, and Supplementary Fig. 2a,b) and structures (insets of Fig. 2a,b and Supplementary Fig. 2a,b) are obtained by either employing epitaxial growth of Ti (Fig. 2a,b – main text), and pulsed laser deposition of TiO_2 (Supplementary Fig. 2a,b). Supplementary Fig. 2c,d shows the corresponding samples in Supplementary Fig. 2a,b after the annealing treatment described above (i.e., 20 h at 450°C, 0.1 mbar O_2). Comparison of large- and small-scale STM images shows that morphology and atomic-scale structure are respectively retained even after such a harsh treatment. Minimal morphology changes, comparable to those described in the main text, occur only for the $SrTiO_3(110)$–(2 × 5) structure, which appears somewhat rougher after annealing, with the appearance of a few, single-atomic-layer-high islands. It should be stressed that, while STM imaging allows to confirm that the $SrTiO_3(110)$–(4 × 1) and –(2 × 5) structures are stable at the atomic and mesoscopic scales, an analogous conclusion can be extended to the macroscopic scale by analysis of the LEED patterns of the samples (bottom-right insets of Supplementary Fig. 2). In particular, several LEED patterns on the whole surface of the sample (not shown here) have been acquired by macroscopically moving the sample in the electron beam. All LEED images acquired on the two parts of the surface bi-crystal showed analogous structures at each step of the preparation.

Several SIMS depth profiles (Supplementary Fig. 3) were measured on such a surface bi-crystal after the ^{18}O isotope exchange treatment described above, i.e., 16 h anneal in $^{16}O_2$, followed by 4 h anneal in $^{18}O_2$. The first measurement point is excluded. As in the main text, the surface exchange coefficient, k^*, was determined from

$$k^* = \frac{j}{(f_{\text{out}} - f_{\text{bg}})\, c_{\text{O}}} \,. \qquad \text{(Supplementary Eq. 1)}$$

Supplementary Eq. 1 includes the ^{18}O flux density j, the tracer fraction $f_{\text{out}} = 0.971$ (i.e., the ^{18}O fraction in our isotope-enriched exchange gas), the background ^{18}O fraction f_{bg}, approximated as the natural abundance of ^{18}O (i.e., $f_{\text{bg}} = 0.00205$), and the concentration c_{O} of oxygen in $SrTiO_3$.

With the assumption of constant exchange rate during the exchange period t, the tracer flux density can be expressed as

$$j = \frac{M}{t} \,, \qquad \text{(Supplementary Eq. 2)}$$

where M represents the area-specific total amount of incorporated tracer

$$M = \frac{1}{A} \int c_{^{18}\mathrm{O}}(z)\, \mathrm{d}V\,. \qquad \text{(Supplementary Eq. 3)}$$

In Supplementary Eq. 3 $c_{^{18}\mathrm{O}}(z)$ is the volume concentration of exchanged tracer, and A the cross-sectional area measured by SIMS, while z represents the depth coordinate. Since each SIMS measurement has been performed on uniformly-terminated regions of the $SrTiO_3$ bi-crystal, the volume integral in Supplementary Eq. 3 can be reduced to an integration along the depth coordinate, yielding

$$\begin{aligned} M &= \int c_{^{18}\mathrm{O}}(z)\, \mathrm{d}z = c_\mathrm{O} \int \left(f_\mathrm{m}(z) - f_\mathrm{bg}\right) \mathrm{d}z \\ &= c_\mathrm{O} \sum_{\substack{\text{measured}\\ \text{points}}} \left(f_\mathrm{m} - f_\mathrm{bg}\right) d_\mathrm{s}\,. \end{aligned} \qquad \text{(Supplementary Eq. 4)}$$

In Supplementary Eq. 4, the ^{18}O tracer concentration $c_{^{18}\mathrm{O}}$ is expressed in terms of the oxygen concentration in $SrTiO_3$, c_O, and the isotope fraction exceeding the background measured in SIMS. Moreover, the continuum depth integration is substituted by a summation over the discrete SIMS measurement points, times the sputter depth per measured point d_s.

Inserting Supplementary Eqs. 2 and 4 into Supplementary Eq. 1 yields the expression used for the determination of the effective surface exchange constant in the present work

$$k^\star = \sum_{\substack{\text{measured}\\ \text{points}}} \frac{\left(f_\mathrm{m} - f_\mathrm{bg}\right) d_\mathrm{s}/t}{\left(f_\mathrm{out} - f_\mathrm{bg}\right)}\,. \qquad \text{(Supplementary Eq. 5)}$$

As for the profiles presented in the main text, direct fitting of the depth profiles measured after 4 h ^{18}O exchange did not yield reliable results due to their extremely shallow extension of only a few nanometers. In such a depth range, SIMS-related profile broadening effects cannot be neglected, as discussed in Supplementary Note 3 below.

The values of $k^\star$ for 1 h and 4 h annealing periods, respectively, show no significant trend depending on the duration of the annealing treatment [$\sim$ $(4-6) \times 10^{-15}$ m s^{-1} for (2×5)–, and $\sim$ $(1-2) \times 10^{-14}$ m s^{-1} for (4×1)–reconstructed surfaces, respectively], and the very same ratio of effective surface exchange constants $k^\star_{(4\times1)}/k^\star_{(2\times5)}$ is observed in both experiments, namely $k^\star_{(4\times1)}/k^\star_{(2\times5)} = 3.1 \pm 0.3$, and $k^\star_{(4\times1)}/k^\star_{(2\times5)} = 3.1 \pm 0.6$ after 1 h, and 4 h annealing periods, respec-

tively. This means that, in good approximation, the incorporation over time via the surface remains constant, at least over several hours. We can thereby exclude that either saturation effects or different oxygen transport in $SrTiO_3(110)$ near-surface regions are responsible for the observed differences between the two reconstructions. Consequently, only the different activity of the respective surface structures is responsible for the observed effects.

Supplementary Note 3

Experimental broadening of SIMS profiles

Broadening of depth profiles occurs in SIMS due to atomic mixing as a consequence of the sputter ions used for depth profiling (Cs^+ ions, 2 kV, $\sim$ 100 nA in the present study), and of the impact and implantation of the primary ions (Bi_3^{++} clusters, 25 kV, $\sim$ 0.02 pA)[2,3]. As a result, the isotope composition attributed to deeper atomic layers is affected by the composition of the higher-lying ones. Such an effect is of minor importance when isotope profiles with diffusion lengths larger than 10 nm are investigated. However, the SIMS results reported in Fig. 3b (main manuscript) and Supplementary Fig. 3 show depth profiles rapidly decaying towards the natural abundance of ^{18}O within a few nanometers.

In order to address if these ^{18}O depth profiles are effectively representative of diffusion profiles, or if they are rather dominated by broadening effects, Supplementary Fig. 4 compares the shape of the decay of the ^{18}O concentration on (4×1)– and (2×5)–reconstructed areas. In the former case, given that (i) both reconstructed areas belong to the very same bulk crystal, and (ii) we measure no difference in band-bending/surface potential by XPS – meaning that the whole sample is characterized by a uniform 'bulk' diffusion constant – the difference in k^* values between the two surface structures should lead to a different decay length, and, as a result, to different profile shapes. Conversely, in case readily-decaying profiles are dominated by SIMS broadening effects, the very same shape as a function of depth should be observed. As can be observed in Supplementary Fig. 4a, the rescaled ^{18}O concentration profiles measured on (4×1)– and (2×5)–reconstructed areas after short annealing times (1 h) are essentially superimposed, indicating that such profiles are dominated by SIMS broadening effects. Upon longer annealing periods (4 h, Supplementary Fig. 4b) the decay lengths of the ^{18}O concentration profiles on (4×1) and (2×5) are still similar, but no longer virtually identical. The visible difference arises in particular from some broadening of the profile in the (4×1)–reconstructed area. Therefore, we conclude that the decay of these latter depth profiles at

least partly originates from true tracer diffusion dynamics.

However, a strong indication that SIMS-related broadening effects still significantly contribute to the data in Supplementary Fig. 4b comes from the comparison of the scaled profiles measured on (2×5)–reconstructed regions (Supplementary Fig. 4c), whose shape appears essentially unaffected by the four-fold increase in exchange duration. This observation strengthens the conclusion that on $SrTiO_3(110)$–(2×5) areas oxygen exchange involves exclusively very near-surface layers, on a length scale shorter than the depth resolution of SIMS, consistently with the findings of DFT calculations (see main manuscript, and Supplementary Notes 7 and 8). For this reason, and since comparable decay lengths are observed for all depth profiles in Supplementary Fig. 4, SIMS-related broadening of the ^{18}O profiles are important also for long annealing times (Supplementary Fig. 4b). As a result, fitting such profiles – as it is customarily done in these cases – using Fick's diffusion law does not yield satisfactory results, and provides unreliable values for k^* and D^* coefficients. Therefore, only k^* values have been extracted from the total ^{18}O amount incorporated, as described in Supplementary Note 2.

Supplementary Note 4

Work function of $SrTiO_3(110)$–(4×1) and –(2×5) surfaces

In order to assess any difference in work function between (4×1)–, and (2×5)–reconstructed $SrTiO_3(110)$ surfaces, x-ray-excited (Al Kα, $h\nu = 1486.61$ eV) secondary electron emission spectra were measured at a surface bi-crystal (Supplementary Fig. 5). The bi-crystal was prepared as detailed in the main manuscript. It should be stressed that this approach allows to unequivocally measure work function differences solely arising from the two $SrTiO_3(110)$ reconstructions, independently of any extrinsic influence.

We measured the secondary electron cutoff energy, which can be straightforwardly related to the sample work function[4]. The electron emission spectra were acquired at normal emission, by negatively polarizing the sample with a 9 V (nominal) battery. Such a precaution prevents unwanted cutoffs due to the detection system, conferring the electrons a minimum kinetic energy higher than any analyzer work function (4–5 eV). To compensate for this bias voltage, and therefore allowing to determine absolute values of work functions rather than mere differences, O 1*s* core-level spectra were acquired on both regions, with the sample held at both ground and at the polarization potentials. This allows for a correction of the horizontal energy axis in Supplementary Fig. 5. In

order to minimize electric-field-lines distortion, which can significantly affect work function measurements[4], the x-ray source was significantly retracted from the surface (approximately 50 mm). All measurements were performed overnight, to limit the effect of any stray, time-varying magnetic fields. Subsequent spectra acquired on both regions show a deviation in the measured cutoff smaller than the standard error of the fitted position.

The secondary electron cutoff was evaluated by fitting a sigmoidal step function to the main, lowest-kinetic energy peak in each spectrum. The cutoff positions were evaluated as the energies corresponding to 5% of the step amplitude, resulting in work functions of (4.051 ± 0.010) eV and (4.470 ± 0.003) eV for $SrTiO_3(110)$–(2 × 5) and –(4 × 1) surfaces, respectively. Uncertainties derive from the combined standard errors on the fitting parameters of the sigmoidal step, and the position of the O 1*s* core levels.

Additional features in the spectra of Supplementary Fig. 5 are possibly related to low-energy Auger emission, and were not present on secondary emission spectra measured on the sample plate. Their characterization is beyond the scope of the present manuscript.

Supplementary Note 5

XPS core level positions of (4 × 1)– and (2 × 5)–$SrTiO_3(110)$

Supplementary Fig. 6 reports core-level x-ray photoelectron spectra (Al Kα, $h\nu = 1486.61$ eV) acquired on the $SrTiO_3(110)$ surface bi-crystal described in the main manuscript. Clearly the features corresponding to electron emission from O 1*s*, Ti 2*p*, and Sr 3*d* are all found at the same binding energy (within the experimental uncertainty of ± 0.03 eV). This indicates that no difference exists between the surface potentials of (4 × 1)– (black), and (2 × 5)–reconstructed (red) $SrTiO_3(110)$ surfaces. Once more it should be stressed that the present measurement, performed on the very same bulk sample on which both surface structures are present at the same time, allows to unequivocally disentangle the structure-related surface potential from extrinsic effects deriving from, e.g., the bulk of the single crystalline sample, or the detailed configuration of the electric contact with the measuring set-up.

Supplementary Note 6

V_O concentration estimates

As detailed below, according to defect chemical models in the literature[5–7], at our experimental conditions the nominal equilibrium concentration of V_Os in 0.5 wt.% Nb-doped $SrTiO_3$ is approximately 6×10^{-7} cm^{-3}, or about 10^{-29} with respect to oxygen sites. However, it is possible that this thermodynamic lower limit of oxygen vacancies is not reached within our experiments. Therefore, here we also determine an upper limit of oxygen vacancy concentration by using an estimate of the decay length of the tracer profiles.

Electron-conducting donor-doped $SrTiO_3$ may undergo a re-oxidation process when exposed to high temperatures[6], accompanied by a complex time dependence of the corresponding defect concentration profiles due to the changing band bending during re-oxidation. SrO evolves at the surface, and at equilibrium Sr vacancies largely compensate the donor dopants. Since our samples were annealed at about 1000°C in 3×10^{-9} bar O_2 for 1 h, such a re-oxidation may have taken place also in our case, at least in the relevant region close to the surface. With the database of Ref. 6 we can estimate the resulting equilibrium defect concentrations for the given pressure and temperature: An oxygen vacancy concentration of about 5×10^{14} cm^{-3} and an electron concentration of about 5×10^{18} cm^{-3} result. Hence, a remaining effective donor concentration of 5×10^{18} cm^{-3} is found, corresponding to those Nb donors still uncompensated by Sr vacancies.

This cation defect chemical state is 'frozen' while cooling the sample after annealing. Possibly some minor cation changes take place also during cooling, and the frozen-in temperature is thus somewhat lower than 1000°C; this may further reduce the effective donor concentration very close to the surface, but the following conclusion is not affected by this uncertainty: Before the diffusion experiment, the $SrTiO_3$ sample was exposed to 0.1 mbar oxygen at 450°C for about 4 h and the oxygen stoichiometry equilibrates according to these conditions. In contrast to annealing at 1000°C, however, all cations – and thus also Sr vacancies – can be considered immobile at this temperature[6]. As a consequence, the equilibration takes place without changing the effective donor concentration of about 5×10^{18} cm^{-3}. From the defect chemical data in the literature[6] we then get a nominal oxygen vacancy concentration of 6×10^{-7} cm^{-3}, i.e., virtually all oxygen vacancies that were formed at 1000°C should become refilled at 450°C during oxygen exposure. This is also in accordance with our experimental observation that no oxygen vacancies are observed on the $SrTiO_3$ surfaces by STM.

However, as discussed in Supplementary Note 3 the tracer diffusion profile of the (4×1)–

reconstructed $SrTiO_3(110)$ surface seems to broaden to a small extent after 4 h diffusion time, compared to the 1 h exchange period, i.e., at least some oxygen vacancies seem to exist. An upper limit of this concentration can be determined from an estimated decay length L_D in the profile of about 2 nm. With $L_D \approx \sqrt{D^\star t}$ we find $D^\star = 3 \times 10^{18}$ cm^2 s^{-1} . Using data[6] of the vacancy diffusion coefficient D_V we get from $D^\star = 0.69\, D_V\, C_V$ an upper limit of the vacancy concentration C_V of about 6×10^{12} cm^{-3}, or a vacancy fraction with respect to oxygen sites of 10^{-10} (possibly with a spatial variation within the diffusion zone).

Supplementary Note 7

DFT formation energy for V_Os at (4 × 1) and (2 × 5) surfaces

Several vacancy sites were inspected in the surface (S), subsurface (S-1) and interface region (I) between the reconstructed overlayer and the bulk-like $SrTiO_3$ substrate, as indicated in Supplementary Fig. 7. The results, collected in Supplementary Table 1, reveal that the formation of V_O in the (2 × 5) surface layer is by 2.2 eV more favorable than at the very surface of the (4 × 1) reconstruction. We note that the DFT slab effectively models the (2 × 4) member of the (2 × n) family; to avoid any confusion with the experimental results we adopt the notation (2 × 5). As already reported in Ref. 8 we find that the energetically more advantageous V_O site in the (4 × 1) is in the interface layer.

Supplementary Note 8

O_2 adsorption and dissociation: first principles molecular dynamics

The first principles molecular dynamics (FPMD) runs delivered several final structures for the process describing the adsorption and dissociation of O_2 on both types of surface reconstructions. First, in our MD runs we did not start from a 'true' gas phase (i.e., O_2 many ångstroms above the surface), as this would have required a much larger vacuum region and much longer MD runs. Considering the large size of our supercells, this would have been computationally too costly. To model the adsorption and dissociation process we have performed several MD runs starting from different initial configurations with the O_2 molecule located at different positions/orientations at about 2 Å above the surface. Depending on the initial configuration, we had cases in which the O_2 landed on the surface and dissociated, and cases in which the O_2 desorbed back in the vacuum region. The structures involving both adsorption and dissociation turned out to be by far the energetically most

stable configurations. In the main text, we show the most representative ones. In Supplementary Fig. 8 we have collected a few other models representing (a) dissociation of O_2 on the large hollow area of the (4×1) surface ($E_{ads} = -2.94$ eV), (b) O_2 dissociation on the defect-free (2×5) surface along the Sr-chain ($E_{ads} = -0.87$ eV), and (c) the unfavorable (positive formation energy) situation of O_2 adsorption in the middle of the (2×5) surface with one V_O. Supplementary Table 2 summarizes all the DFT-calculated O_2 adsorption energies for the configurations reported in the main text and in this Supplementary Information.

Supplementary Note 9

O_2 dissociation paths and barriers calculated by nudged elastic band (NEB)

Supplementary Figure 9 shows the NEB paths discussed in the main text, and reports the initial, transition and final states, the corresponding activation energy (i.e., the one at the transition state), and the overall energy gain in the final state.

Supplementary Note 10

Polyhedral flexibility: phonon softening

The degree of flexibility of a polyhedron is quantifiable by the softness of the lowest phonon mode that is responsible for structural deformation or rotations. A similar concept has been used by Li and Benedek[9] for quantifying the flexibility of polyhedra in assisting oxygen interstitial diffusion. To prove further the generality of the concept of polyhedral flexibility, we have performed phonon calculations on AO_x clusters for A = Ti, Cr and V and $x = 6, 5, 4, 3$. The structures have been obtained by performing full structural relaxations using a quasi-Newton algorithm without any symmetry constraint, starting from the AO_6 phase and progressively removing one oxygen atom from the optimized AO_x to form an initial AO_{x-1} structure. The phonon frequencies and dynamical matrix for the optimized structures were obtained using finite differences, and the force constants have been computed with the help of the phonopy package[10]. The resulting optimized structures and the force constants for the lowest (i.e., less stable) phonon mode are collected in Supplementary Fig. 10.

These results clearly show that the rotational phonon in the AO_5, AO_4, and AO_3 polyhedra exhibit much more negative (i.e., unstable) modes compared to that in the AO_6, confirming that the perovskite AO_6 environment is the preferred and the most rigid one. The increased flexibility of the

unsaturated polyhedra with respect to the octahedral environment is also explained by the larger degree of asymmetry of the unsaturated clusters in terms of the A–O–A bond-angles, which are significantly altered in the unsaturated clusters as compared to the 90° configuration in the AO_6 phase. In AO_5 the out-of-plane A–O–A angle is reduced to 85°, in AO_4 the A–O–A angles are increased to 109°, and in AO_3 the three A–O–A angles measure 120°. The average Ti–O bond length, on the other hand, decreases monotonically from 1.89 Å in TiO_6 to 1.78 Å in the TiO_3 cluster (qualitatively similar trends are obtained for VO_x and CrO_x clusters).

Supplementary Note 11

Surface phase diagram with defects or adsorbates

The final defect structures obtained by combining chemical intuition and extensive molecular dynamic runs should represent stable phases. However, a phase diagram of the most stable phases would help the interpretation of the results and its connection with the observations. Following the standard recipe and prescriptions of *ab-initio* thermodynamics[11–13] (described in detail also in Ref. 14), which we have previously used for $SrTiO_3$,[14] we have constructed the surface phase diagram as a function of the O and Ti chemical potentials for the two types of $SrTiO_3$ reconstructions under scrutiny, specifically the (4 × 1) and (2 × 5), considering the clean surfaces and the defected ones (with one O_2, and with one O_2+V_O). The results shown in Supplementary Fig. 11 clearly indicate that the five phases show areas of stability for specific ranges of the Ti and O chemical potentials. The only phase that does not appear in the phase diagram is (2 × 5) + (O_2+V_O), i.e., the less stable one according to our results reported in Fig. 4 of the main text. Nonetheless, by inspecting the relative stability of the (2 × 5) + (O_2) and (2 × 5) + (O_2+V_O) phases at $\Delta\mu_{Ti} = -4$ eV and $-1 > \Delta\mu_O > 0$ – corresponding to the region of stability of (2 × 5) + (O_2) – we find that these two phases are very close in energy, about 30 meV Å^{-2}, as shown in Supplementary Fig. 12.

Supplementary Methods

Image processing. All STM images in the main text and in the present Supplementary Information have been post-processed by ImageJ. To all images a first-order (small-scale images) or second-order (piezoelectric creep on large area images) background has been subtracted. Minimal removal of line-jumps has also been employed when needed (e.g., tip change). Thermal-drift and piezo-creep correction was applied in post-processing of the images. LEED images were cropped to one-quadrant without loss of information, and are displayed in inverted gray scale. Specific references to filenames of raw data are reported in the figures, where applicable.

Spectra processing. No post-processing was used for spectroscopy results, except for area/height normalization where explicitly indicated. Analysis of LEIS/XPS/WF spectra and SIMS profiles was accomplished by OriginPro. Raw, non-smoothed data are presented throughout. For SIMS depth profiles, the first measurement point was excluded from analysis and representation, since this is usually seriously affected by the presence of unavoidable contamination when transferring samples trough air.

DFT structure rendering. The DFT structures have been rendered with POV-Ray (with the exception of Supplementary Fig. 8, for which VESTA was employed).

Supplementary References

[1] Gerhold, S., Riva, M., Yildiz, B., Schmid, M. & Diebold, U. Adjusting island density and morphology of the $SrTiO_3(110)$-(4×1) surface: Pulsed laser deposition combined with scanning tunneling microscopy. *Surf. Sci.* **651**, 76–83 (2016).

[2] Liu, Y., Hofmann, S., Wang, J. Y. & Chakraborty, B. R. Quantitative reconstruction of Ta/Si multilayer depth profiles obtained by time-of-flight-secondary-ion-mass-spectrometry (ToF-SIMS) using Cs^+ ion sputtering. *Thin Solid Films* **591**, 60–65 (2015).

[3] Hofmann, S., Liu, Y., Jian, W., Kang, H. L. & Wang, J. Y. Depth resolution in sputter profiling revisited. *Surf. Interface Anal.* **48**, 1354–1369 (2016).

[4] Helander, M. G., Greiner, M. T., Wang, Z. B. & Lu, Z. H. Pitfalls in measuring work function using photoelectron spectroscopy. *Appl. Surf. Sci.* **256**, 2602–2605 (2010).

[5] Denk, I., Münch, W. & Maier, J. Partial conductivities in $SrTiO_3$: Bulk polarization experiments, oxygen concentration cell measurements, and defect-chemical modeling. *J. Am. Ceram. Soc.* **78**, 3265–3272 (1995).

[6] Meyer, R., Zurhelle, A. F., De Souza, R. A., Waser, R. & Gunkel, F. Dynamics of the metal-insulator transition of donor-doped $SrTiO_3$. *Phys. Rev. B* **94**, 115408 (2016).

[7] Saraf, S., Markovich, M. & Rothschild, A. Defect chemistry of *pn* junctions in complex oxides. *Phys. Rev. B* **82**, 245208 (2010).

[8] Wang, Z. *et al.* Anisotropic two-dimensional electron gas at $SrTiO_3(110)$. *Proc. Natl. Acad. Sci. U. S. A.* **111**, 3933–3937 (2014).

[9] Li, X. & Benedek, N. A. Enhancement of ionic transport in complex oxides through soft lattice modes and epitaxial strain. *Chem. Mater.* **27**, 2647–2652 (2015).

[10] Togo, A. & Tanaka, I. First principles phonon calculations in materials science. *Scr. Mater.* **108**, 1–5 (2015).

[11] Bottin, F., Finocchi, F. & Noguera, C. Stability and electronic structure of the (1×1) $SrTiO_3(110)$ polar surfaces by first-principles calculations. *Phys. Rev. B* **68**, 035418 (2003).

[12] Reuter, K. & Scheffler, M. Composition, structure, and stability of $RuO_2(110)$ as a function of oxygen pressure. *Phys. Rev. B* **65**, 035406 (2001).

[13] Reuter, K. Ab initio thermodynamics and first-principles microkinetics for surface catalysis. *Catal. Lett.* **146**, 541–563 (2016).

[14] Wang, Z. *et al.* Vacancy clusters at domain boundaries and band bending at the $SrTiO_3(110)$ surface. *Phys. Rev. B* **90**, 035436 (2014).
