# Supplementary material for: Influence of surface atomic structure demonstrated on oxygen incorporation mechanism at a model perovskite oxide
Source: Nat Commun. 2018 Sep 13;9:3710. doi: 10.1038/s41467-018-05685-5 (PMC6137039; doi:10.1038/s41467-018-05685-5)
Supplement: Supplementary file 2 — Description of Additional Supplementary Files [file 41467_2018_5685_MOESM2_ESM.pdf]

Description of Additional Supplementary Files

File Name: Supplementary Data 1
Description: Archive (.zip) containing the DFT-calculated structures presented in the main text and in the Supplementary information. The files are provided in .cif format. The archive is organized in the following folder tree:

- Main Manuscript: DFT structures shown in Figures 1 and 4 of the main text. Subfolders "Figure1" and "Figure4" contain the corresponding .cif files.
- Supplementary Information: DFT structures reported in Supplementary Figures 7, 9, and 10. Subfolders "SupplFig07", "SupplFig09", and SupplFig10" contain the corresponding .cif files. "SupplFig09" further contains one sub-folder for each panel of Supplementary Figure 9."
